# Materials and techniques used for the “Vienna Moamin”: multianalytical investigation of a book about hunting with falcons from the thirteenth century

**DOI:** 10.1186/s40494-021-00553-w

**Published:** 2021-07-23

**Authors:** Wilfried Vetter, Bernadette Frühmann, Federica Cappa, Manfred Schreiner

**Affiliations:** Institute of Science and Technology in Art, Academy of Fine Arts Vienna, Schillerplatz 3, 1010 Vienna, Austria

**Keywords:** Illuminated manuscripts, Non-invasive, Material analyses, Painting technique, Scanning XRF, Reflection FTIR, Raman, FORS, Mosaic gold, Brazilwood lake

## Abstract

A multianalytical approach was used to characterize the materials in the “Vienna Moamin”, an outstanding richly illustrated manuscript from the late thirteenth century, which was made in Italy and is now kept in the Kunsthistorisches Museum Wien. The investigations were carried out with a non-invasive approach by using complementary techniques, such as X-ray fluorescence (XRF), reflection Fourier transform infrared spectroscopy (rFTIR), Raman spectroscopy, and fiber optic reflectance spectroscopy (FORS). In addition, XRF scans were performed in two areas which yielded chemical maps showing the elemental distribution. The results revealed that typical materials from the medieval times were applied for the manuscript. Calcium carbonate on the parchment surface indicated a dehairing process with lime and/or whitening with chalk. Two different iron gall inks were detected in the main text and marginal notes, and vermilion was used for rubrication. The color palette included azurite, a green colorant composed of orpiment and indigo, yellow ochre, brown iron oxide pigments, minium, vermilion, brazilwood lake, and carbon black. Moreover, mosaic gold was detected in gold-beige hues. Lead white was identified for white areas and fine decoration lines, as well as in mixture with blue and red pigments for light color shades. No reliable information could be obtained concerning the binding media. Two differing application techniques for gold leaf were detected, which correspond with stylistic differences: either on gypsum or chalk preparation layers. Furthermore, calcium soap contents in certain colors were determined only on one folio with unique characteristics. The XRF scans of two historiated initials revealed that similar materials were applied in both cases and provided further valuable information about the painting technique. The results obtained enabled to gain insights into Italian thirteenth century manuscript production techniques and to characterize the used materials. The investigations showed the importance of scanning XRF for the elucidation of painting techniques, but also the demand of scanning devices utilizing compound specific analytical techniques such as rFTIR.

**Supplementary Information:**

The online version contains supplementary material available at 10.1186/s40494-021-00553-w.

## Introduction

**Fig. 1:**
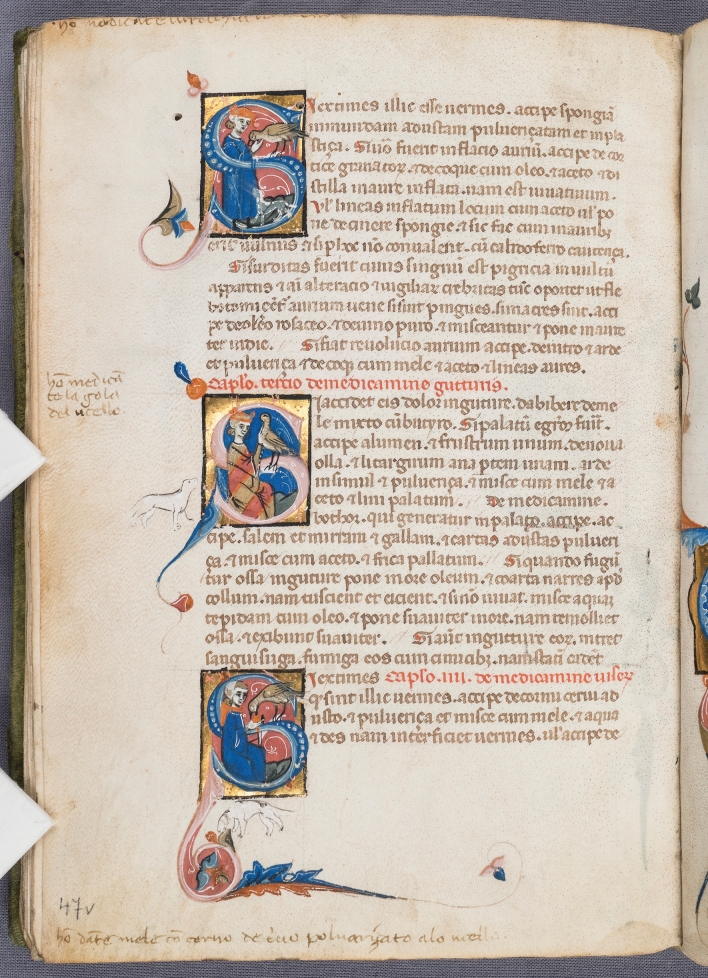
Visible light image of folio 47v in the Vienna Moamin (thirteenth century)

In the year 1240 Emperor Frederick II of Hohenstaufen (1194–1250), a great enthusiast of falconry and author of an unrivalled treatise on the subject, “De arte venandi cum avibus”, ordered the translation into Latin of an Arabic treatise based on texts of the eighth and ninth century. His court philosopher Theodore of Antioch thus compiled the “Liber Moamin falconarii de scientia venandi per aves et quadrupedes” (The Book of Falconer Moamin on the Art of Hunting with Birds and Quadrupeds), which was personally revised by Frederick II during the siege of Faenza [1]. The Latin text became famous and was copied multiple times, and it was also translated into Neapolitan, Tuscan and Franco-Italian [2]. The “Vienna Moamin”, a luxury volume for a high ranking person, is one of the 29 surviving Latin copies, and dates back to circa 1300. At the middle of the fifteenth century it was in Hungary, in the hands of Janos Rozgonyi, member of the royal court, before being acquired by a Viennese humanist and physician, Johannes Fuxmagen who wrote his name in the volume. At the beginning of the sixteenth century the manuscript was part of the collection of Emperor Maximilian I (1459–1519), a passionate hunter himself, and later on it passed to Archduke Ferdinand II. of Tyrol (1529–1595) [2]. It is nowadays kept in the Imperial Armoury (Hofjagd- und Rüstkammer), Kunsthistorisches Museum Wien (Inv. No. K4984). The manuscript was made in central or southern Italy by presently unknown artists and consists of 54 parchment folios with single column text and 101 historiated initials. Interestingly, instructions in Italian for the illuminator were written in the margins and have survived for the most part, giving an insight into the creation of the scenes [3]. As an example, folio 47 verso is presented in Fig. 1. The parchment and the text of the manuscript are relatively well preserved. The same accounts for the majority of the illuminations, although delamination of gold leaf and localized paint losses occurred frequently. Extensive media loss must be observed only on few folios, including folio 1r which was investigated in this study. It represents a document of high cultural and historical significance, which contains an in-depth treatment of the species of birds of prey used in falconry and the medical treatment of their illnesses, as well as those of hunting dogs. A real gold and a standard facsimile edition have been brought out recently [4]. Moreover, a commentary volume including codicological information is presently in press [5].

Progresses in the design of mobile analytical instruments, which enable to characterize materials in manuscripts non-invasively in libraries and museums, led to an increased collaboration between natural sciences and the humanities, particularly material sciences and philology. A common interest concerns the question if the results from material analyses can be used to attribute manuscripts to particular authors or workshops. This seems basically possible, as the materials in medieval times often contained impurities or characteristic trace elements. Moreover, the application techniques were not standardized and specific practices of certain workshops could provide useful information for an attribution. However, it is not as simple in practice. An unambiguous attribution requires ideally complete data sets containing the chemical and historical information about local workshops/persons over long time periods, which may serve as references for the particular styles. For this reason, one aim of the Centre of Image and Material Analysis in Cultural Heritage in Vienna (CIMA) [6] is to build corresponding databases as complete as possible and to link it with other institutions in a further step.

To the best of our knowledge, only few chemical data has been published concerning the materials in late thirteenth century manuscripts from Italy [7]. Hence, the results reported in this study may add to the knowledge about medieval manuscript production and provide reference data for the attribution of other manuscripts. Some obstacles which may complicate an attribution, e.g. the contribution of multiple artists, have been mentioned by Trentelman and Turner [8] and will be addressed as well in the present study. Moreover, the strengths and weaknesses of the applied analytical techniques will be discussed. Particular attention will be given to XRF scanning, which has been increasingly used in combination with other techniques for the study of manuscripts in the last years [9–12].

## Experimental

During two measurement campaigns the Vienna Moamin was investigated at the Academy of Fine Arts Vienna (2017) and the conservation workshop of the Collection of Arms and Armour, Kunsthistorisches Museum Wien (2018) by using mobile equipment for completely non-invasive analyses. The applied techniques were X-ray fluorescence (XRF), Raman spectroscopy, reflection Fourier transform infrared spectroscopy (rFTIR), and fiber optic reflectance spectroscopy (FORS).

**Fig. 2:**
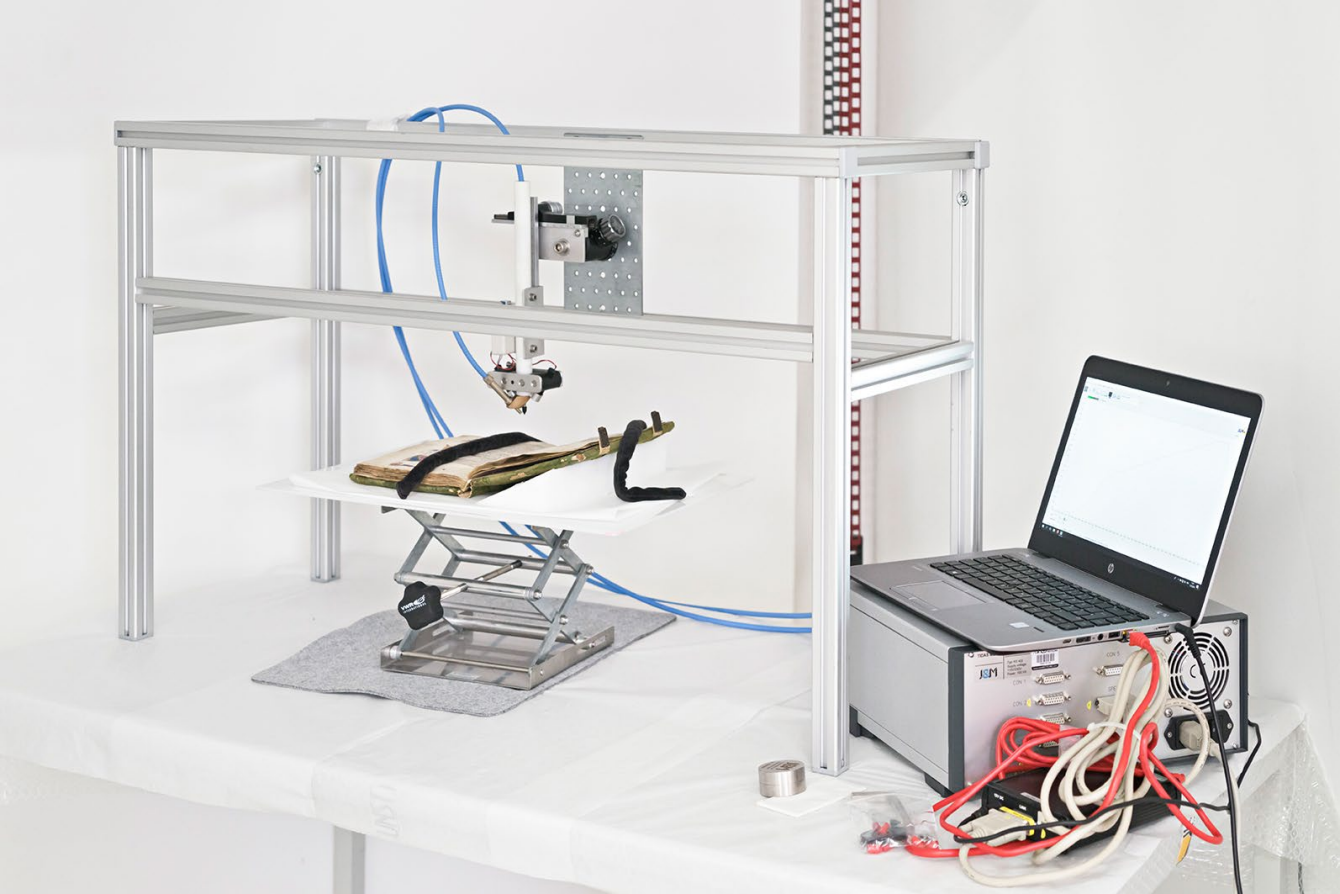
FORS analyses of the Vienna Moamin. A lifting platform was used for the adjustment of the measuring position and the adequate distance. The fiber optic probe was mounted on a tetrapod, which also served to mount the rFTIR instrument

During the analyses the manuscript was placed horizontally on a lifting platform, which was used for adjusting the measuring position and distance. A felt below the lifting platform facilitated its horizontal movement. The instruments were mounted either on a tripod for the XRF and Raman analyses, or a tetrapod in case of rFTIR and FORS as shown in Fig. 2. The tetrapod provides maximum stability, particularly if the rFTIR instrument weighing ca. 8 kg is mounted, and can be disassembled for easy transport.

Prior to the analyses, 21 folios were chosen after visual inspection of the manuscript with the intention to cover as many analytical questions regarding materials and techniques as possible. Due to the limited access to the manuscript, not all measurement points were analyzed by all methods available. The analyzed folios are listed in Table 1. In total 186 measurement points (mp) were analyzed. XRF was applied on 21 folios (171 mp), rFTIR on 10 folios (66 mp), Raman on 11 folios (36 mp), and FORS on 5 folios (19 mp). XRF scanning was performed on folios 10v and 47v.

**Table 1:** List of folios analyzed in the Vienna Moamin

Technique	Folio										
	1r	1v	3r	3v	10v	16v	17v	19r	23r	26v	27r
XRF	×	×	×	×	×	×	×	×	×	×	×
rFTIR	×				×	×		×	×		
Raman	×	×	×	×	×	×					
FORS	×				×			×			
XRF scanning					×						

Technique	Folio									
	35r	37r	37v	44v	47v	48v	49r	49v	51v	52r
XRF	×	×	×	×	×	×	×	×	×	×
rFTIR		×	×		×	×		×		
Raman		×	×	×	×				×	
FORS		×			×					
XRF scanning					×					

Images of folios 1r, 10v, 19r, 37r, and 47v which also show the analyzed points are provided in Additional files 1, 2, 3, 4 and 5.

### XRF single point measurements

The manuscript was examined with a spectrometer Type ELIO (XGLab S.R.L./Bruker Nano Analytics, Milano, Italy). It consists of a 4 Watt X-ray rhodium (Rh) tube and a silicon drift chamber detector (SDD) with a resolution of 155 eV and an active area of 25 mm^2^. The measuring geometry is 0°/63.5° with an X-ray spot of 1 mm diameter. The excitation voltage was set to 40 kV with a tube current of 60 µA, and the acquisition time was 60 s. The system was operated with software version 1.6.0.29, which was also used for the evaluation of the data.

### XRF scanning

Two historiated initials were scanned by using the ELIO spectrometer in steps of 0.5 mm in x and y direction and an acquisition time of 2 s per measurement point (mp). The excitation voltage was set to 40 kV with a tube current of 100 µA. Two elemental maps were captured on folios 10v and 47v, which consisted of 40 × 60 mps (scanning time 80 min) and 85 × 85 mps (scanning time 240 min). The software includes a mapping application, which allows a manual definition of the keV range for the elements to be mapped.

### rFTIR

A spectrometer Alpha (Bruker Optics, Ettlingen, Germany) equipped with a DTGS detector and a module for external reflection was available for the rFTIR measurements. An image of the IR spot obtained by an Osiris camera for infrared reflectography (Opus Instruments, Norwich, UK) showed that it covers ~ 5 mm (roughly circular), with increasing intensity towards the center. However, Legrand et al. reported an experimentally determined effective spot size of 1.8 mm (horizontal) and 2.1 mm (vertical) [13]. The background was acquired using a gold mirror and reflectance spectra (average of either 64 or 128 scans) were collected in the range between 7000 and 375 cm^−1^ with a resolution of 4 cm^−1^. The operation of the instrument and the evaluation of the spectra was performed with Opus 7.5 software. The reflectance spectra were evaluated either without processing, or occasionally after calculation of absorption index spectra by Kramers–Kronig transformation in the range between 4000 and 375 cm^−1^, followed by baseline correction (concave rubberband correction, 10 iterations, 64 baseline points). The available spectral databases included reflectance spectra of materials in the material collection of the Institute of Science and Technology in Art, Academy of Fine Arts Vienna, as well as spectra measured in transmission mode from the IRUG 2007 database [14].

### Raman spectroscopy

Raman analyses were performed with a micro-Raman spectrometer Pro-Raman-L-Dual-G (Enwave Optronics, Irvine, CA, USA) equipped with a 785 nm Diode Laser (~ 350 mW), a fiber optic probe with 50 × long working distance objective (Leica Microsystems, Wetzlar, Germany), and a Peltier cooled two dimensional CCD array detector. The spot size was 50 µm and spectra were collected in the range between 3000 and 100 cm^−1^ with a resolution of 8 cm^−1^. RamanReader data collection software was applied to operate the system and the spectra obtained were compared to reference spectra from the collection of samples by using Opus 7.5.

### Fiber optic reflectance spectroscopy (FORS)

FORS analyses were conducted using a spectrometer MSP400 (J&M Analytik AG, Aalen, Germany) equipped with a halogen lamp, a 244 diode array detector, and quartz fiber optics. The self-built probe [15] has a 0°/45° measuring geometry and a collimator enables a spot diameter of 1.5 mm (see Additional file 6). Reflectance spectra were collected either in the range between 380 and 900 nm or 320 and 1147 nm. The spectrometer was operated utilizing TidasDAQ3 software. Additionally, Panorama 4.0 software was used for spectra evaluation by comparison to a database with spectra obtained in materials from the collection of samples mentioned above.

## Results and discussion

### Materials used to make the Vienna Moamin

A summary of the basic materials used by the scribes and illuminators for the Vienna Moamin including the results of the respective analytical techniques is given in Table 2 and the results are discussed more in detail subsequently.

**Table 2:** Materials determined by XRF (including XRF scanning), rFTIR, Raman, and FORS in the Vienna Moamin. Minor elements determined by XRF are presented in brackets

Material	Conclusion	XRF	rFTIR	Raman	FORS
**Carrier material for writing and painting**					
Whitish folios	Parchment with CaCO_3_ content	S, Cl, K, Ca, (Fe)	Parchment, calcium carbonate	Calcium carbonate	Uncharacteristic
**Writing inks**					
Brown, main text	Iron gall ink	Fe, (Cu, Pb)	Uncharacteristic partially calcium oxalate	Iron gall ink	Increasing reflectance in NIR
Brown, marginal notes	Iron gall ink	S, K, Fe, (Cu)	Uncharacteristic	Iron gall ink	Not analyzed
Red ink, main text	Vermilion	S, Hg	Uncharacteristic	Vermilion	Vermilion
**Colorants**					
White	Lead white	Pb	Lead white	Not analyzed	Uncharacteristic
Blue	Azurite	Fe, Cu	Azurite	Not analyzed	Azurite
Light blue	Azurite, lead white	Fe, Cu, Pb	Azurite, lead white	Not analyzed	Azurite
Green	Orpiment, indigo, gypsum	Si, S, K, (Fe), As	Gypsum	Orpiment & indigo	Indigo
Gold-beige	Mosaic gold	S, Sn	Uncharacteristic partially gypsum	Not analyzed	Not analyzed
Yellow	Ochre, kaolin, lead white	Si, (Ti), Fe, Cu, Pb	Kaolin, lead white	Not analyzed	Not analyzed
Orange	Minium	Pb	Parchment bands attenuated	Minium	Not analyzed
Red	Vermilion	Hg, S	Uncharacteristic	Vermilion	Vermilion
Red	Brazilwood lake	Ca	Calcium carbonate	Not analyzed	Brazilwood lake
Pink	Brazilwood lake, lead white	Ca, Pb	Calcium carbonate, lead white	Not analyzed	Brazilwood lake
Brown	Iron oxide	Ca, Fe	Uncharacteristic	Iron oxide	Not analyzed
Brown	Iron oxide with silicate	Si, S, Ti, Fe	Not analyzed	Not analyzed	Not analyzed
Black	Carbon black	Only elements from parchment	Uncharacteristic	Carbon black	Carbon black

In general, our results suggest that the color palette includes colorants which have been widely used for book decoration throughout the Middle Ages, e.g. lead white, azurite, minium or vermilion. On the other hand, materials such as mosaic gold or Brazilwood lake seem to have been used not before the late thirteenth century—the time when the Moamin was manufactured. Interestingly, Nabais et al. [16] report an almost similar color palette compared to the Moamin (except the additional use of lapis lazuli) in a Galician-Portuguese songbook (The Ajuda Songbook) dated around 1300.

### The parchment

The detection of calcium carbonate (CaCO_3_) by rFTIR and Raman on the parchment surface argues for the use of lime for dehairing of the skins, as residues of lime may react with atmospheric CO_2_ to form CaCO_3_ [17] and/or whitening of the parchment with chalk. The bands with maxima at 1450 cm^−1^ and 875 cm^−1^ in the absorption index spectra match with the IRUG reference calcite MP00108, and were assigned to ν_3_-asymmetric CO_3_ stretching and ν_2_-asymmetric CO_3_ deformation, respectively [18]. Additionally, calcium soaps were determined by rFTIR in a single measurement point on folio 37r, which possibly represents a reaction product of residual subcutaneous fat from the skins with calcium carbonate. The main fatty acids in animal fat are oleic acid, palmitic acid, and stearic acid [19], which all show relatively similar FTIR spectra [20]. The characteristic bands of calcium stearate are observed at 2957 cm^−1^ (antisymmetric ν_a_CH_3_), 2922 and 2852 cm^−1^ (antisymmetric ν_a_CH_2_ and symmetric ν_s_CH_2_), as well as 1580 and 1546 cm^−1^ (antisymmetric ν_a_COO^−^ stretching in unidentate and bidentate coordination with calcium ions) [21]. Calcium soaps were also determined in various other manuscripts during our investigations [6], e.g. in Codex slavicus 8 (fourteenth century, Austrian National Library), but the non-invasive techniques used did not allow to receive further information concerning the origin of the material.

### Brown and red writing inks

**Fig. 3:**
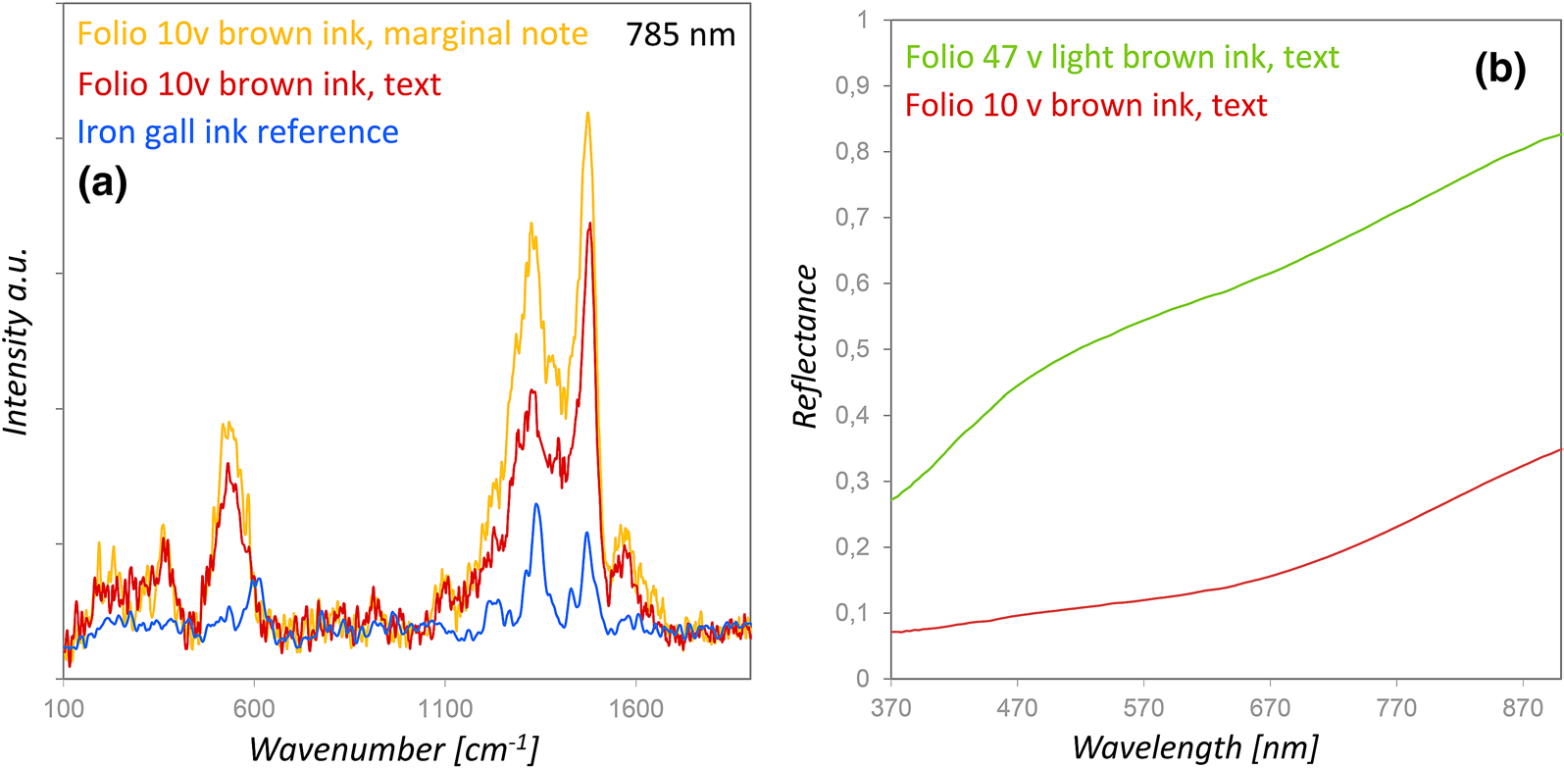
**a** Comparison of Raman spectra of the brown ink in the text (red) and the marginal note (yellow) on folio 10v with an iron gall ink reference spectrum (blue) and **b** a FORS spectrum of the brown ink in the text of folio 1v compared with the light brown ink (green) on folio 47v

XRF analyses revealed different elemental compositions of the brown inks used for the main body of the text (Fe with low Cu and Pb contents) and the marginal notes (S, K and Fe with low Cu content). In both cases the Raman spectra showed the characteristic spectral features of iron gall inks, which were, according to the literature [22–24], at about 1576, 1478 (strong), 1329 (strong), 535, and 373 cm^−1^ (Fig. 3a). FORS spectra of the brown inks of the main text showed increasing reflectance values towards the NIR region (Fig. 3b) analogously to the iron gall ink spectra reported by Aceto and Cala [25].

No indications of iron gall inks were obtained by rFTIR. The relatively large spot size of the rFTIR instrument did not allow an analysis of the ink alone without the surrounding parchment, which contributed to the spectra obtained. However, a comparison of several measurement points in the main text to the blank parchment showed increased intensities in the absorption index spectra in the regions of the amide I (vCO, vCN and δNH) and the ωCH_2_/δCH (methine) parchment bands at 1645 cm^−1^ and 1334 cm^−1^ [26]. These features overlap with the antisymmetric ν_a_CO and symmetric ν_s_CO vibrations of calcium oxalate dihydrate (weddellite) [27] and match with the IRUG reference spectrum weddellite MP00425. The formation of oxalates in iron gall inks indicates oxidative degradation of organic compounds [28], although the mechanisms are currently not well understood.

Vermilion was identified in the red writing inks, which was proved by XRF, Raman and FORS. Characteristic for semiconductor pigments, the FORS spectra of vermilion showed a sharp S-shaped increase of reflectance starting from ca. 550 nm and an inflection point at ca. 600 nm. The characteristic elements were Hg and S, although the XRF Kα line of S (2.305 keV) is coinciding with Pb Mα- (2.346 keV) and Hg Mα-lines (2.195 keV) in two spectra from folios 3r and 37v. However, these two measurement points were not analyzed by complementary methods and it remains unclear, whether the Pb contents resulted from an addition of minium to the inks or from a material transfer from surrounding areas containing minium or lead white. The spectra obtained by rFTIR were similar to the spectra measured on the bare parchment, as vermilion only absorbs at wavenumbers below the range available with the spectrometer used (375 cm^−1^) [29].

### Blue

**Fig. 4:**
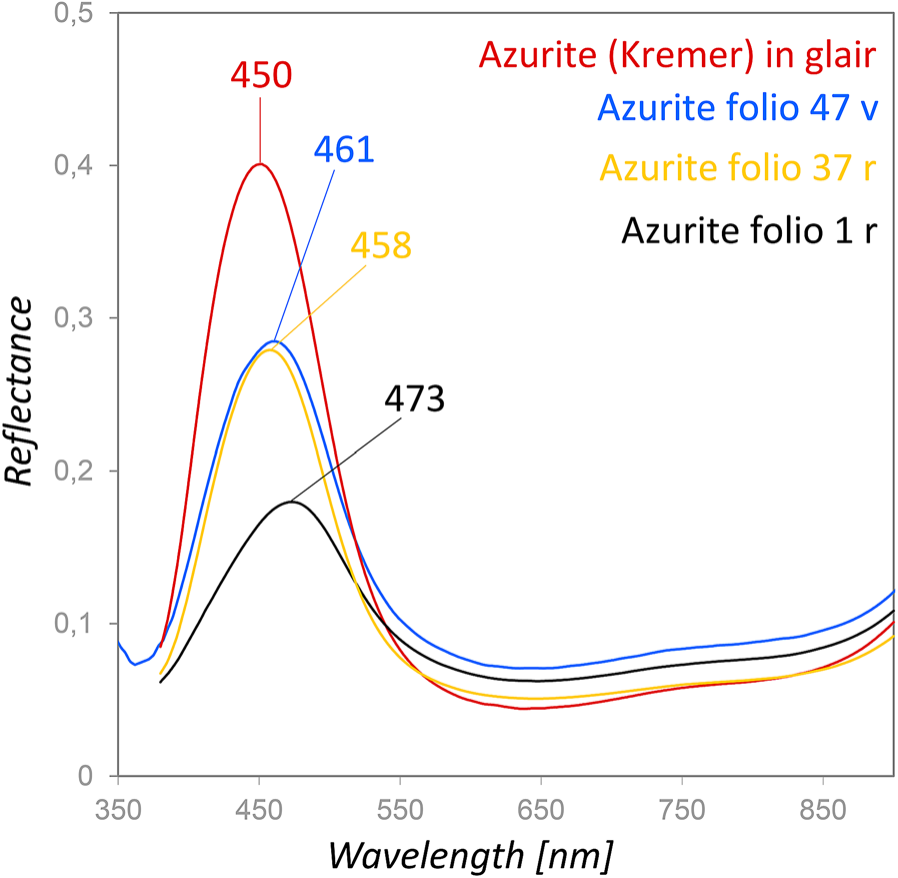
Comparison of the FORS spectra of azurite (Kremer 1673112) (red) with measurement points on folio 1r (black), 37r (yellow) and 47v (blue) shows differing reflectance maxima in the blue spectral region

The identification of azurite (Cu_3_(CO_3_)_2_(OH)_2_) in the Vienna Moamin has already been published in an rFTIR study discussing the characteristic spectral features of the pigment mixed with various binding media [30]. XRF detection of Cu supported the rFTIR results. In addition, smaller quantities of Fe and Si indicate typical impurities of mineral azurite, particularly hematite and silicates [31], although no evidence for silicates could be derived from the rFTIR spectra. Additionally, Pb was detected by XRF in most of the measurement points in the blue areas, which mainly derives from fine white decorative lines over the blue ground layer (Fig. 1). Various reflectance maxima were observed in the FORS spectra (Fig. 4), particularly 473 nm on folio 1r, 458 nm on folio 37r, and 461 nm on folio 47v, compared to 450 nm in case of a contemporary natural azurite pigment (Kremer, order number: 1673112). The blue on folio 1r appears greenish and dull in comparison to the other examples due to the lower reflectance at the maximum as well as its shift to higher wavelengths.

A mixture of azurite and lead white could be determined in the light blue areas, as detected on folio 37r by XRF (Cu, Pb) and rFTIR. The bands in the fingerprint region of the absorption index spectra match with the IRUG references azurite MP00001 and lead white MP00107 (see Additional file 7). Furthermore, two characteristic bands at 4378 cm^−1^ (overtone 3ν_3_) and 4244 cm^−1^ (combination ν + δ O–H) in the rFTIR spectrum enable an easy identification of azurite [30]. The ν_3_ ${\text{CO}}_{3}^{{2 - }}$ asymmetric stretching vibration of azurite at around 1400 cm^−1^ overlaps with the analogous feature of lead white. However, lead white was identified preferably in the absorption index spectrum by means of the sharp ν_4_ ${\text{CO}}_{3}$ in-plane bending vibration at 683 cm^−1^ and the weaker ν_1_ ${\text{CO}}_{3}$ symmetric stretching vibration at 1045 cm^−1^ [32].

### Green

**Fig. 5:**
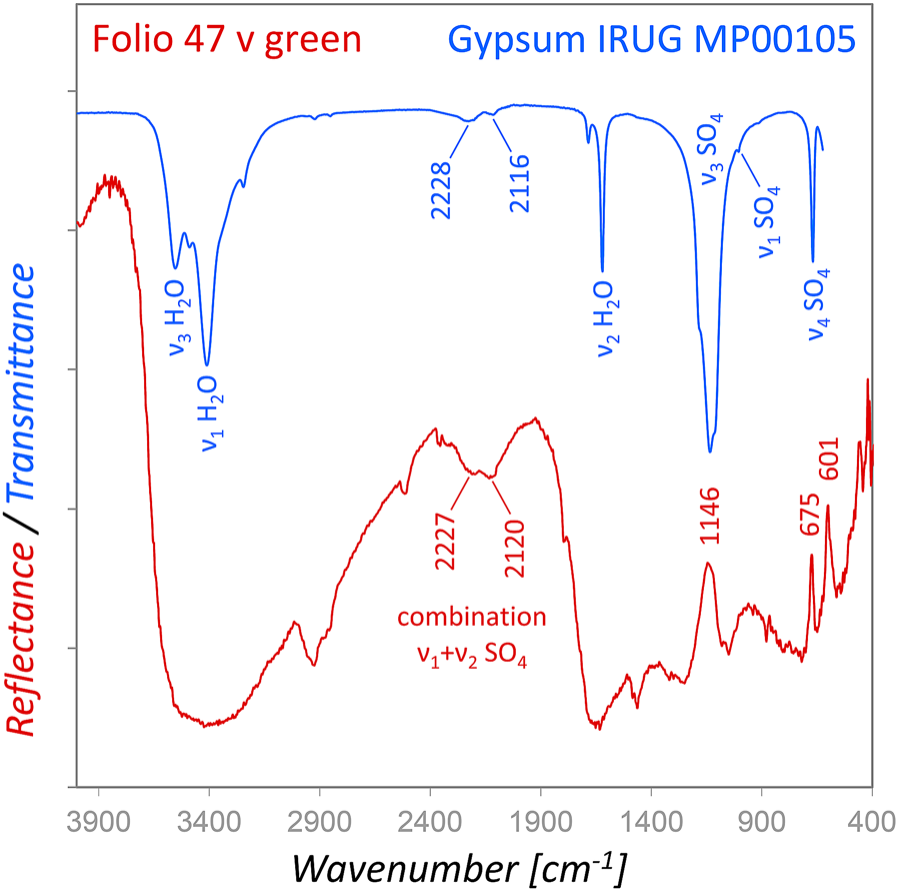
Comparison of the reflectance spectrum obtained from a green area (red) with a reference spectrum of gypsum measured in transmission (blue). The strong SO_4_ vibrations (ν_3_ and ν_4_) are inverted, but the OH bands are almost invisible

Green areas in the historiated initials and decorations were painted by using a mixture of indigo and orpiment. Orpiment was detected by XRF (As, S) and Raman spectroscopy, whereas the characteristic spectral features of indigo matched with the respective Raman and FORS reference spectra (see Additional file 8). The indigo content was too low for a determination by rFTIR, but the spectra showed the characteristic features of gypsum. It has been reported [33, 34] that gypsum often is associated with orpiment derived from geothermal or volcanic environments and hence, it can be considered most probably as an impurity of the yellow pigment. The characteristic bands of gypsum were inverted in the rFTIR spectra showing maxima at ca. 1146, 675, and 601 cm^−1^, corresponding to the ν_3_ SO_4_ and ν_4_ SO_4_ vibrations [35]. The inversion of bands is often observed in reflection FTIR spectra of oxyanion pigments and results from the specularly reflected proportion of the radiation [36]. Moreover, combination bands of ν_1_ and ν_3_ modes of SO_4_ at 2227 and 2120 cm^−1^ appear similar to transmission spectra, e.g. IRUG reference gypsum MP00105, and the OH bands were generally almost invisible in the reflectance spectra for unknown reasons (Fig. 5).

### Yellow

The elements Si, Ti, Fe, and Pb were detected in a yellow color solely on folio 1r by means of XRF, which suggests the use of yellow ochre. Ti is a minor element, the Kα line is very weak and the Kβ line is imperceptible. The content of Ti and other minor elements (K, Ca) is characteristic for individual ochres and is used for their classification [37, 38]. However, we did not quantify the Ti content and K and Ca could not be determined due to the relatively high contents of both elements in the parchment. The rFTIR results as well as the image from the on-board camera suggested that Pb derives from fine decoration lines painted with lead white, which have partially flaked off. Silicon is a main constituent of kaolinite. Si–O stretching bands (between 1130 and 970 cm^−1^) as well as Al–O–Si deformation (537 cm^−1^) and Si–O–Si deformation (467 cm^−1^) bands [39] are inverted in the reflectance spectrum. Furthermore, OH stretching bands in the corresponding absorption index spectrum at 3690, 3669 and 3617 cm^−1^ match with the IRUG kaolinite reference MP00109.

### Gold-beige

The color was often applied in the historiated initials, as well as ornamental decorations. It appears moderately shiny. In three single measurement points, Sn and Pb were detected by XRF, although in various ratios. This would, in principle, argue for the use of lead tin yellow (Pb_2_SnO_4_). In fact, an in-depth evaluation and interpretation of the XRF scanning data, which will be discussed below in the respective section, showed that mosaic gold (SnS_2_) was used in this manuscript. The rFTIR results indirectly supported this finding, as no characteristic spectral features could be observed in the region between ca. 580 and 400 cm^−1^, where Nodari and Ricciardi [40] reported inverted bands in the rFTIR spectra of lead tin yellow type 1 in egg white at 569, 504, 459, and 427 cm^−1^. In the rFTIR spectra the parchment bands were strongly attenuated by the pigment layer and mainly very broad spectral features were observable. In addition, the spectrum obtained on folio 37r showed the characteristic vibrations of gypsum ν_3_ SO_4_ at 1141 and 1119 cm^−1^, as well as ν_4_ SO_4_ at 669 and 594 cm^−1^, though relatively weak. According to the literature, mosaic gold, also known as aurum musicum or purpurinus, is a golden-brown pigment with a very subdued sparkle, which was first used in the thirteenth century, but still was considered a novelty by the treatise writers in the fourteenth century [41, 42]. In this respect, the detection of mosaic gold in the Moamin is an example of a quite early use of mosaic gold. Nabais et al. [16] also consider the detection of mosaic gold in the Galician-Portuguese Ajuva Songbook dated around 1300 as an early occurrence in manuscripts. Unfortunately, the gold-beige color was not analyzed by Raman spectroscopy or FORS, which also would be appropriate methods for the identification of the pigment. The reason therefore is that the Raman analyses mainly focused on the characterization of the writing inks, black and green pigments and FORS mainly on the blue, green and red pigments. We experienced in previous analyses of various manuscripts that particularly inks, as well as black and green pigments can hardly be characterized by XRF, rFTIR or FORS. Furthermore, the limited access to the manuscript did not allow a complete study of the materials by all methods applied. However, the detection of the pigment in manuscripts has been reported by means of a single characteristic Raman band at 314 cm^−1^ [43]. This band can be assigned to a phonon mode of the 2H polytype of SnS_2_ [44], and very similar results were obtained by excitation with either 514 nm or 785 nm lasers [45]. Moreover, a characteristic FORS spectrum with inflection points at 500 and 525–535 nm has been published by Aceto et al. [46]. It should be mentioned that the unique optical characteristics of mosaic gold will surely lead us to use Raman spectroscopy and FORS in addition to XRF for a comprehensive identification in future investigations.

### Orange

Minium (Pb_3_O_4_) was used frequently in initials and decorations. The XRF analysis showed that in orange areas Pb often was detected together with Cu, which most probably derives from a transfer of material from blue areas on the folios with azurite. Minium was also identified by Raman spectroscopy. Three bands in the rFTIR absorption index spectra at 531, 531, and 460 cm^−1^ are in accordance with the data reported for minium measured by using ATR-FTIR [29]. Although the pigment does not absorb in the region between 1700 and 1100 cm^−1^, the bands of parchment are strongly attenuated compared to the spectra of the blank parchment.

### Red and pink

**Fig. 6:**
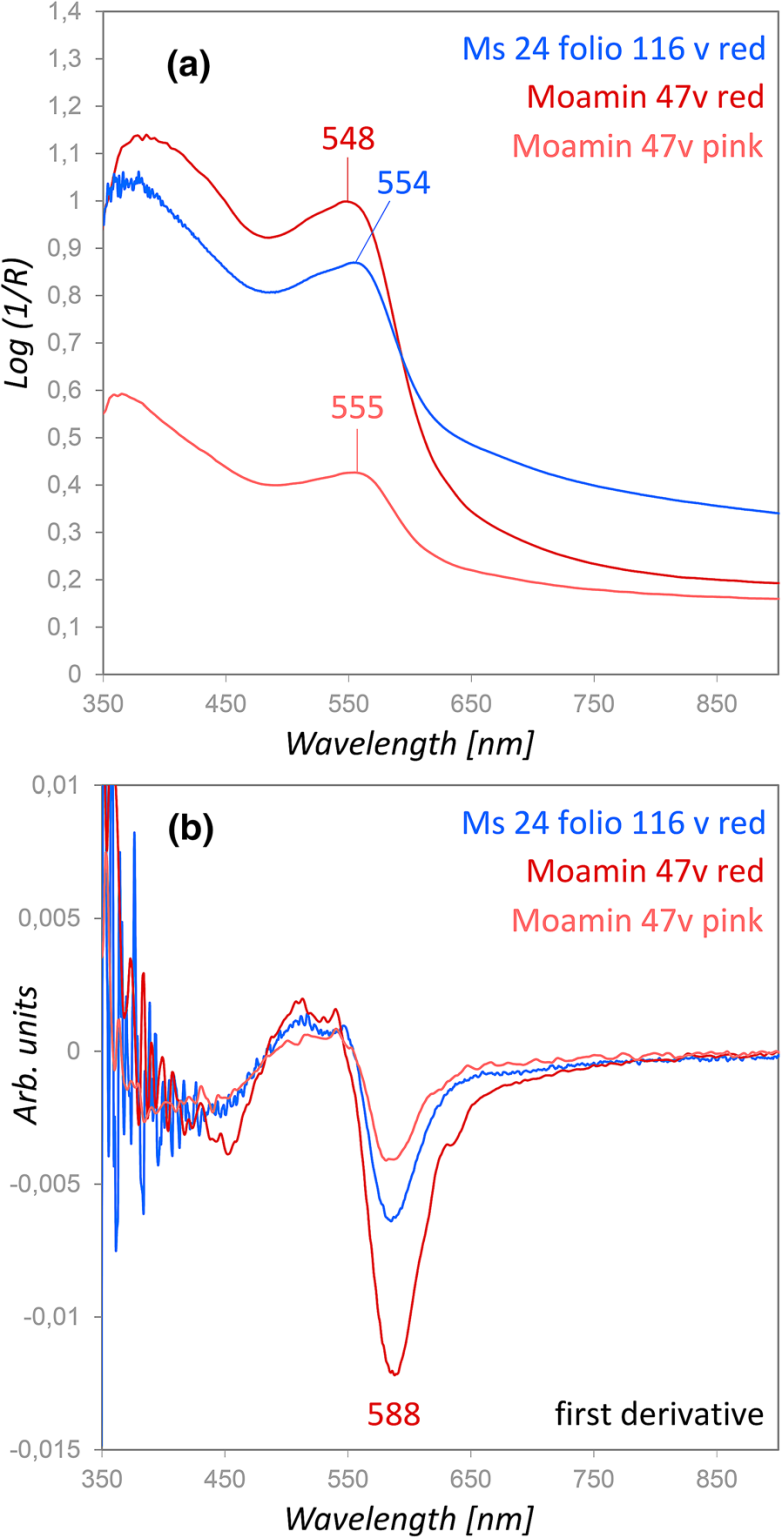
**a** Comparison of the FORS spectra of red and pink areas on folio 47v with a brazilwood lake spectrum measured in a fifteenth century Book of Hours. **b** The first derivative spectra show characteristic inflection points around 588 nm

High amounts of Ca were detected by XRF in the dark red color mainly used in the historiated initials, along with K and few Cu. In accordance with these results the rFTIR analyses showed high quantities of CaCO_3_. However, an identification of the colorant was not possible by these methods. In contrast, FORS analyses indicated the presence of Brazilwood lake, which was further used in combination with lead white for pink hues in the initials. It has been reported that Brazilwood-based colors were used from the fourteenth century [16] and hence, its presence in the Moamin would be an example of an early use. The spectra from a red and a pink area on the middle initial in Fig. 1 (folio 47v) are depicted in Fig. 6a) and show the characteristic spectral features of the Brazilwood chromophore as published by Melo et al. [43] and Aceto et al. [46]. Moreover, a Brazilwood lake spectrum from a fifteenth century Book of Hours (ms 24 in the library of Palalacio Nacional de Mafra (PNM) in Portugal) matches well with the spectra obtained in the Moamin. The spectrum was kindly provided by Tatiana Vitorino, who is co-author of the paper mentioned before. The first derivative of the spectra is shown in Fig. 6b). The inflection points are very close to each other around 588 nm, which further underlines the similarity.

With respect to the identification of CaCO_3_ by rFTIR, Melo et al. [43] reported that CaCO_3_ was frequently added “either to help pigment precipitation or to contribute to the substrate”. The red color was not analyzed by using Raman spectroscopy. However, to our knowledge an identification of Brazilwood lakes in manuscripts by using a 785 nm laser has not been reported so far and even Brazilwood extracts without binding medium seem to produce strong fluorescence with only very weak Raman peaks [47]. Moreover, Mulholland et al. [48] tested various excitation sources (488, 532, 633, 785, 830, and 1064 nm) for the analysis of a Brazilwood watercolor pigment and observed strong fluorescence for any excitation source, and peaks were only observed in case of the 1064 nm laser despite of the high level of fluorescence. This demonstrates the limitations of Raman spectroscopy when only one or two excitation lasers are available.

### Brown

Brown iron oxide was detected on folio 1r by XRF (Ca and Fe) and Raman spectroscopy (see Additional file 9). Moreover, rFTIR revealed an addition of gypsum. In contrast, brown earth pigments with contents of Si and Fe could be determined on other folios.

### Black

In general, the historiated initials were outlined in black and the presence of carbon black was revealed by two broad Raman bands around 1589 and 1324 cm^−1^ in accordance with the literature [49], Additional file 10 shows the respective spectrum. In addition, FORS spectra showed high absorption values in the entire spectral range analyzed. Carbon black was further detected in the depiction of a dog next to the historiated initial on folio 44v (not shown).

### Binding media for illumination

The most important binding media for illumination in medieval times were glair and Arabic gum [50, 51]. In principle, rFTIR would allow the characterization of both materials. In practice the spectra obtained from the paint layers in the illuminations generally showed a contribution of parchment bands, which may overlap with the bands of glair, as both are proteinaceous materials. Hence, no clear statements can be made. Furthermore, we could not detect spectral features of Arabic gum in the colors of the Moamin. A previous study investigating azurite in mixture with glair and Arabic gum suggested that the sensitivity of the used instrument only hardly allows the characterization of binding media [30], which might also account for other pigments.

### Leaf gilding technique

**Fig. 7:**
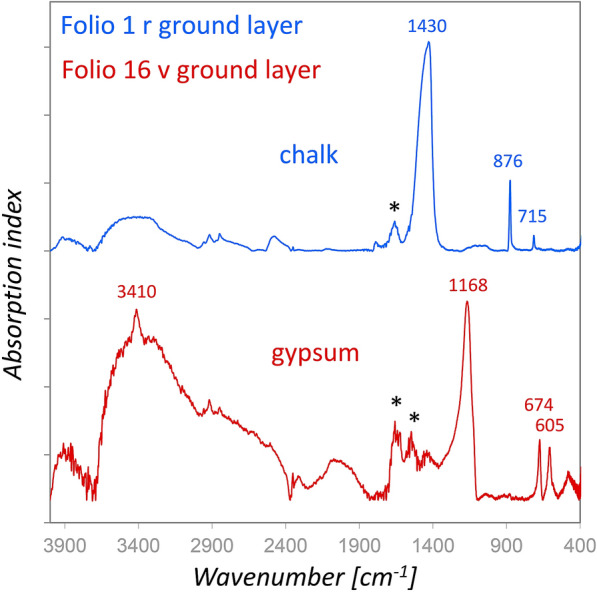
The absorption index spectra from the ground layers for leaf gilding on folios 1r (blue) and 16v (red) show the characteristic bands of chalk and gypsum. The amide I and amide II bands of the proteinaceous binding medium are labelled with black asterisks

Delamination of gold leaf was recognizable on several historiated initials, which enabled a direct analysis of the pink ground layer for the gold leaf on folios 1r, 16v, 19r, and 26v. The rFTIR results showed that calcium carbonate (chalk) was used for the ground layer of folio 1r, whereas gypsum was used on the other analyzed folios (Fig. 7). It is rather probable that the amide I and amide II bands (ca. 1655 and 1555 cm^−1^) in the rFTIR spectra obtained from folio 16v derive from a proteinaceous binding medium than from the parchment below, as the grounds for gilding were applied as relatively thick layers, In accordance with the rFTIR results, solely Ca was detected by XRF on folio 1r, whereas Ca and S were detected on folios 19r and 26v. Sulfur was not unambiguously determined on folio 16v due to a relatively high lead content and hence an overlap of Pb Mα with S Kα.

Furthermore, small intensities Hg and Pb were detected by XRF, indicating that the pink color consists of a mixture of vermilion and a Pb component—minium or lead white—with gypsum. Only inconclusive results were obtained by FORS regarding the colorant. However, the XFR scans on folios 10v and 47v rather suggested that mainly vermilion is present.

**Fig. 8:**
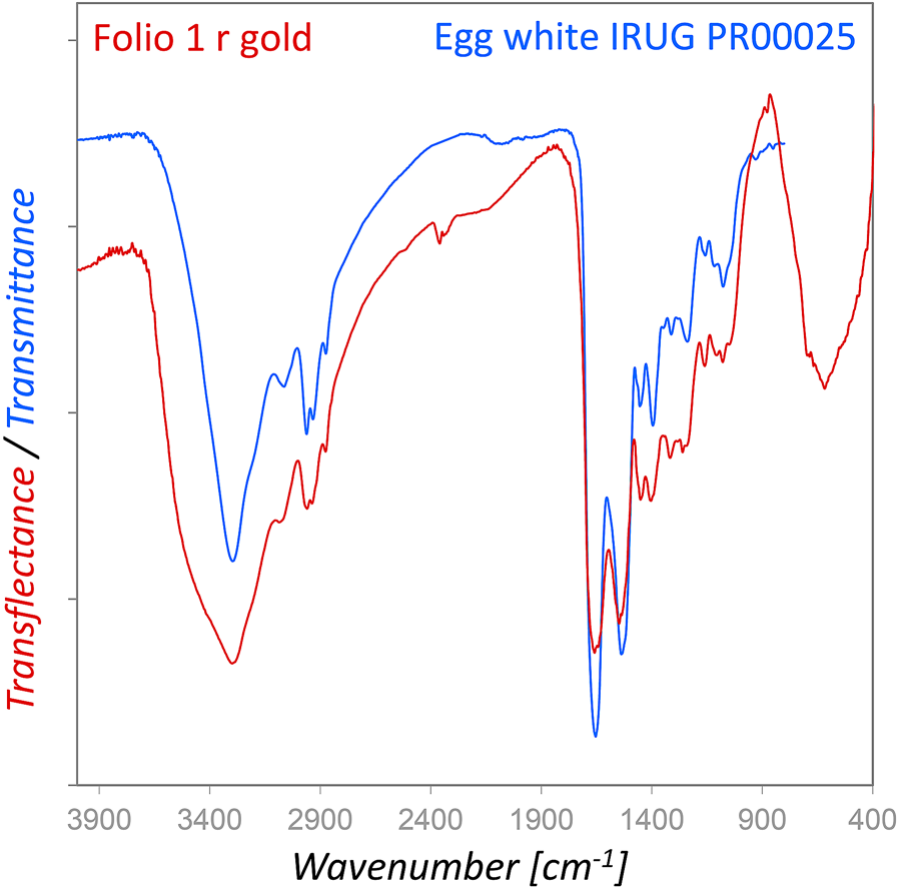
Comparison of the transflectance spectrum obtained from a proteinaceous layer over the gold leaf (red) with the reference spectrum egg white IRUG PR00025 (blue, measured in transmission)

It was interesting to find a proteinaceous layer over the gold leaf on folio 1r and 47v by rFTIR. The gold leaf acted as a mirror for the incident infrared radiation, and hence a transflectance spectrum [52] was obtained. Figure 8 depicts that the spectrum matched well with the IRUG reference spectrum for egg white PR00025, and a comparison with other protein reference spectra showed that glues have much higher amide I/amide II ratios. However, an unambiguous identification of proteinaceous materials by FTIR is not possible.

The proteinaceous compound was further detected on the black outline which partially was applied on top of the gold. It is quite obvious that the results of these single point measurements do not allow statements about the distribution of the material and thus it remains unclear, whether the material represents a layer which was applied onto the gold by intention of the illuminator, an attempt for a consolidation of the badly preserved gilding, or simply a contamination. However, an rFTIR scanning device, as implemented by Legrand et al. [13] would have been useful in order to obtain information regarding these questions.

### The distinct characteristics of folio 1

**Fig. 9:**
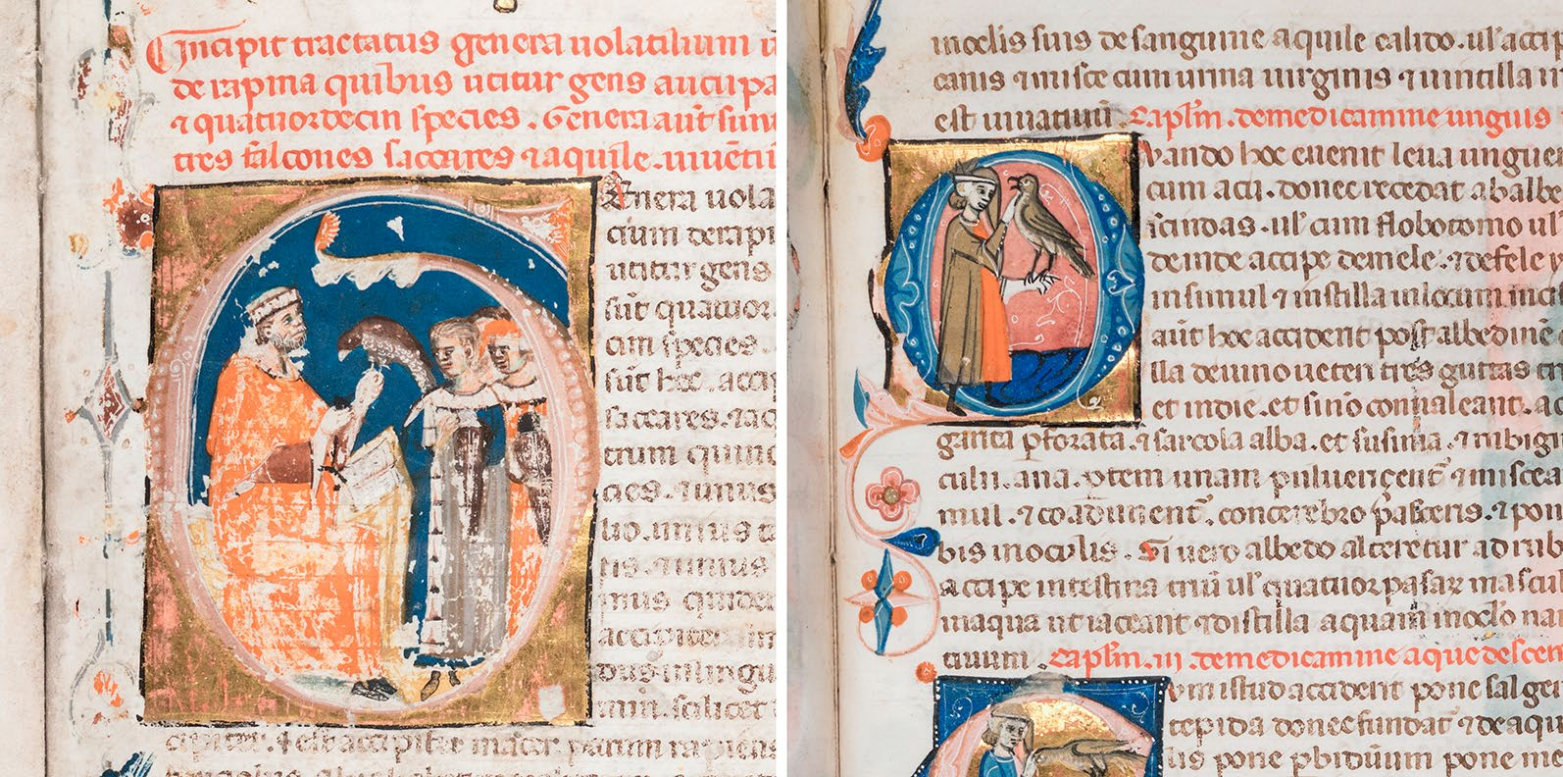
The style of the historiated initial on folio 1r as well as its size differs remarkably from the initials on other analyzed folios such as folio 37r, which is shown as a representative example

It is easily recognizable in Fig. 9 that the historiated initial on folio 1r remarkably differs from most of the other analyzed folios in respect of the size and the depiction of persons and birds. Further examples are provided as Additional files 2, 3, 4 and 5. The same accounts for the artistic design of the other decorations and hence, a different authorship might be assumed. The results of the material analyses clearly supported this assumption. The most remarkable differences include a differing support for leaf gilding (CaCO_3_ instead of CaSO_4_), the use of yellow ochre solely on folio 1r, and contents of calcium soaps in various colors—white, blue, pink, brown, and grey. Interestingly, calcium soaps were not detected in the parchment, the writing red inks, and the yellow or orange areas. Calcium stearate, which is indicative of calcium soaps, can easily be determined by means of the antisymmetric and symmetric C–H stretching vibrations at 2920 and 2850 cm^−1^ and the antisymmetric COO^−^ stretching vibrations at 1575 and 1540 cm^−1^ [21]. A spectrum from a blue color in comparison with the reference spectrum IRUG OF00108 is shown in Additional file 11. Analogously, we observed contents of calcium soaps in certain colors also in Codex slavicus 8 [30], which was manufactured in 1368 in the region Krbava (Croatia). Furthermore, Vieira et al. [53] reported the detection of calcium palmitate in a red colorant (lac dye) on parchment in a Koran dated 1198. This raises the question if soap was added by intention as an attempt to improve the applicability of the color. As noted in the previous section, this question can´t be answered on the basis of single point measurements, which clearly shows a strong demand for rFTIR scanning devices.

### XRF scanning

**Fig. 10:**
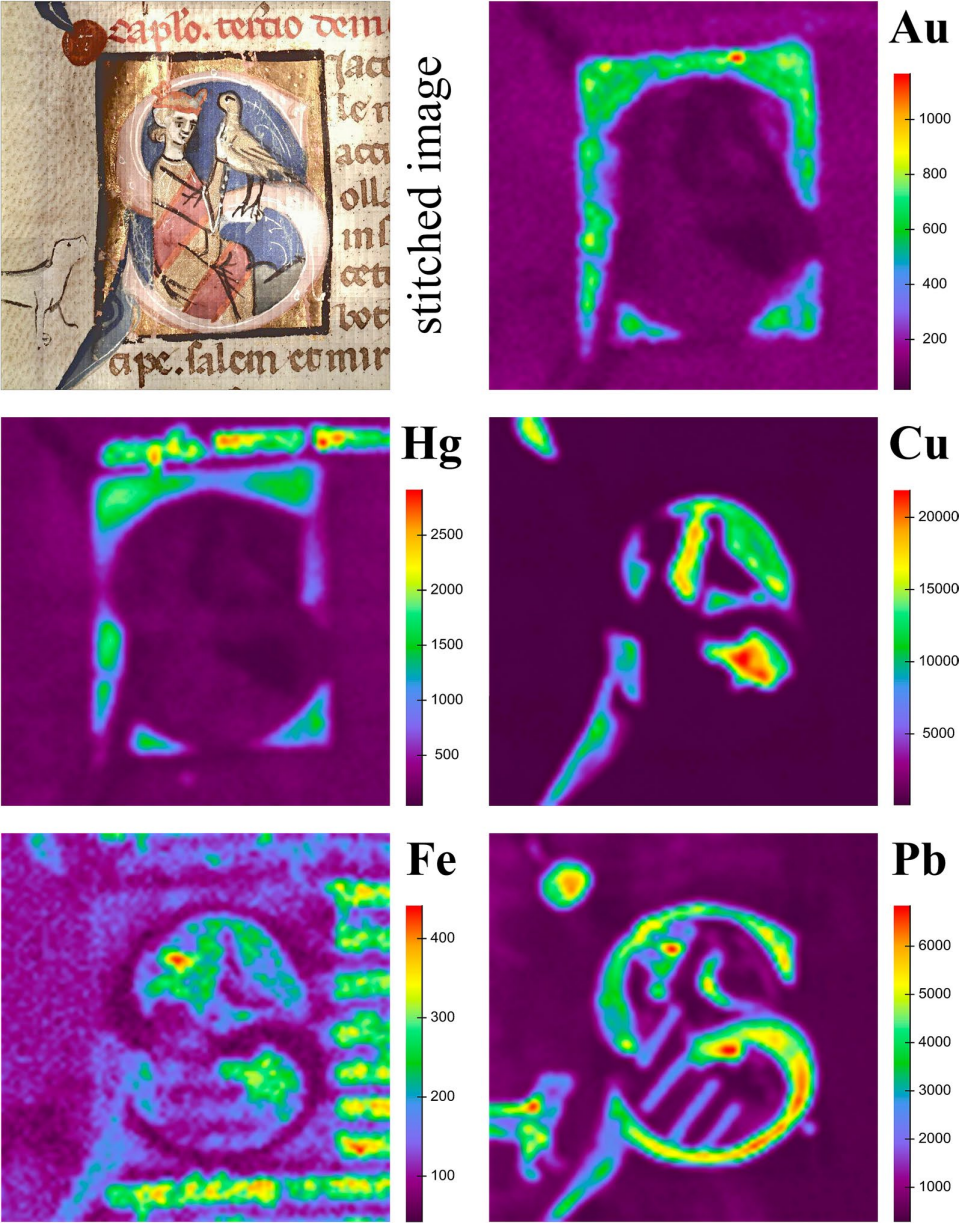
Image stitched by Elio software and the elemental maps of Au, Hg, Cu, Fe, and Pb with the respective intensities (counts) obtained from the historiated initial in the middle of folio 47v

**Fig. 11:**
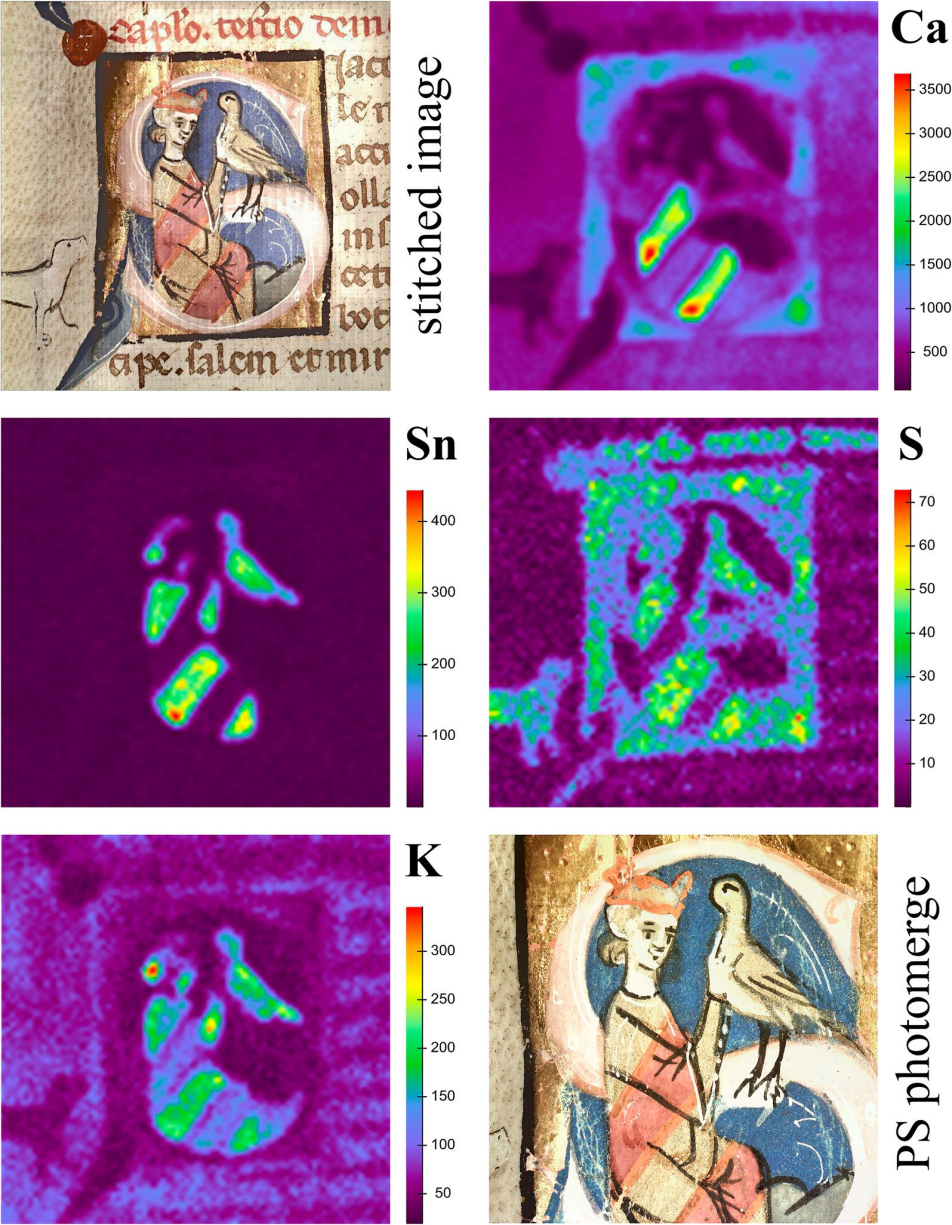
Image stitched by Elio software and the elemental maps of Ca, Sn, S, and K with the respective intensities (counts) obtained from the historiated initial in the middle of folio 47v. A section of the image stitched in Photoshop CS6 is shown on the bottom right

The elemental maps of the historiated initials on folios 10v and 47v yielded substantial information on the painting technique complementing the information obtained by single point measurements and visual light photographs. Both initials were found to be quite similar regarding the painting technique and materials used. Figures 10 and 11 show the results from the scan on folio 47v, particularly the distribution maps of the elements characteristic for the applied colors, which are listed in Table 3.

**Table 3:** Characteristic elements for the colorants used on folio 47v

Element	X-ray emission line (keV range)	Color/material	Occurrence
S	Kα (2.300–2.310) (partial overlap with Pb Mα)	Gold-beige, mosaic gold	Clothing, bird
		Red, vermilion	Writing ink
		Green, orpiment and indigo	Landscape
K	Kα (3.169–3.406)	Gold-beige, together with mosaic gold Sn, S	Clothing, bird
		Red, brazilwood lake	Clothing
Ca	Kα (3.554–3.821)	Red, brazilwood lake	Clothing
		Pink, gypsum	ground layer for leaf gilding
Fe	Kα (6.249–6.485)	Flesh tone, not identified	Falconers incarnate
		Brown, iron gall ink	Writing ink
		Color unclear, together with azurite Cu	Component in blue color
Cu	Kα (7.877–8.144)	Blue, azurite	Background color, decoration
Sn	Lα (3.411–3.484)	Gold-beige, mosaic gold	Clothing, bird
Au	Lα (9.572–9.836)	Gold	Gold leaf
Hg	Lα (10.216–10.720)	Pink, vermilion	Component of ground layer for leaf gilding
		Red, vermilion	Writing ink
Pb	Lα (10.216–10.720)	Red, minium	Clothing, decoration
		Pink, lead white mixed with brazilwood	Initial letter
		Lead white	Dog, decorative lines

Figure 10 shows that Hg (vermilion) is present in the red ink as well as the ground layer below the gold leaf. The low intensity of Pb in the areas with gold leaf indicates that mainly vermilion was used for the pink color of the ground layer for gilding. Pb indicates lead white in the falconers incarnate parts and in the pink colors of the initial letter (mixed with Brazilwood lake), as well as the light blue color in the decoration (mixed with azurite), and moreover, minium in the orange stripes on the clothing. The varying intensities in the Cu map indicates that the thickness of the paint layer is not uniform. Highest Fe intensities are found in the brown iron gall ink, the flesh tone, and also in the blue areas in combination with Cu (azurite). It is rather unlikely, that an iron compound was added intentionally to the blue azurite, as brilliant colors were preferred by the illuminators in the Middle Ages [54]. However, hematite (α-Fe_2_O_3_) and goethite (α-FeOOH) seem to be common impurities in azurite [31].

The quality of the image stitched by the Elio software is rather poor concerning the lateral resolution and color rendering. However, a higher quality stitched image with better color rendering could be obtained when Photomerge in Photoshop CS6 was employed, as depicted on the bottom right in Fig. 11. Additional file 12 shows the original size of the stitched image.

The Ca map shows highest intensities in the red part of the cloth, which was painted with a Brazilwood lake with high contents of calcium carbonate (detected by rFTIR in single point measurements). Lower intensities of Ca were detected in the ground layer for gold leaf, where also S was determined. This indicates that gypsum was used to prepare the ground layer, although a slight contribution of S from vermilion has to be considered, which was added to obtain the pink hue. The coincidence of Sn and S in the elemental maps indicates that the gold-beige color consists of mosaic gold (SnS_2_). Concerning the S map it has to be considered that Pb Mα (2.346 keV) partially coincides with the S Kα (2.305 keV) signal and hence also areas with Pb contents are highlighted. As mentioned above, single point measurements rather argued for the use of lead tin yellow. However, the Pb map in Fig. 11 does not show significant intensities in the gold-beige areas, which strongly argues against the use of lead tin yellow. K was mainly detected in the gold-beige color of the cloths, the bird, and—in lower intensities—in areas with red Brazilwood lake. The role of K in the mosaic gold parts remains unclear since potassium compounds do not seem to have been used for its production according to medieval treatises [55]. Only a thin layer of paint was applied in the green landscape on the bottom right in the initial, where the S map shows relatively high intensities. XRF single point measurements indicated As and S from orpiment in this area. However, it was not possible to visualize the distribution of As due to the overlap of As Kα (10.543 keV) with Pb Lα (10.551 keV) and moreover, the short acquisition time during the scan did not yield enough counts for As Kβ (11.7258 keV) in order to obtain a significant map. These results clearly demonstrate that the acquisition parameters may strongly influence the interpretation of XRF scanning data.

### The illuminators technique of folio 47v

The overall results including visual study from this historiated initial suggest that in a first step a ground layer for gold leaf was applied, consisting of gypsum (Ca), few amounts of vermilion (Hg) and most probably a proteinaceous binding medium. The ground layer solely was applied on areas to be leaf gilded and defines the dimensions of the initial. After the application of gold leaf, the background colors (blue, green), the basic colors of the figural representations (gold-beige, red) and the black outlines were painted. Subsequently, the colors of the initial letters (pink), the decorations emanating from the corners (blue, light blue), and the orange stripes on the clothing were applied, which all partially cover other colors. In the last steps the figural representations were completed with black contour lines as well as white decorative lines (see Additional file 12).

## Conclusions

The Vienna Moamin was investigated non-invasively during two measurement campaigns by using XRF, rFTIR, Raman and FORS. The first campaign aimed to characterize the materials used for this outstanding manuscript, whereas the second campaign mainly focused on the investigation of the painting technique by XRF scanning and answering questions which emerged during the evaluation of the data already obtained. The analyses allowed us to extend our knowledge about the painting techniques and the variety of materials used in the late thirteenth century in Italy. In addition, we detected materials in folio 1r which strongly differed from the materials in the other analyzed folios. This provides analytical indication for a different authorship, so far only assumed by means of stylistic differences.

However, some interesting questions remain unclear, in particular the precise characterization of the gold beige pigment containing Sn, S and K, and also the red Brazilwood lake. Both have not received our increased interest until the evaluation of the data from the second campaign. Unfortunately, no Raman analyses could be performed, which possibly would have enabled a clear identification. This demonstrates the need for improved evaluation tools for the complex spectra obtained from multiple non-invasive analyses, particularly if the access to the studied objects is limited. With respect to the available time for analysis, a microscopic investigation prior to the instrumental analyses probably would have allowed to utilize the available methods more efficient. Moreover, the rFTIR single point measurement detection of a proteinaceous material over the gold leaf, as well as calcium soaps in certain colors raised the question about the distribution of these materials in order to be able to explain the application in the miniature painting technique of the thirteenth century. This strongly indicates an urgent demand for mobile rFTIR scanning equipment, which can be used in libraries or museums.

XRF scanning provided valuable information about the painting technique, although the scans are quite time consuming. In contrast to single point measurements, the risk of misinterpretation of XRF results due to material inhomogeneities is much lower for scans, where the information of multiple measurements is gathered and hence a much clearer picture of the materials can be obtained. This particularly accounted for the gold-beige color, where the detection of Sn and Pb in the single point measurements would rather have argued for the use of lead tin yellow than of mosaic gold. On the other hand, the scanning results showed that the short measurement times for each point may limit the detection of elements present in small quantities, as it was the case for As in the presented study. This clearly shows the importance of single point measurements with longer acquisition times in the areas intended to scan in order to prevent misinterpretation of scan data.

## Supplementary Information

**Additional file 1** Vienna Moamin, folio 1r. The analyzed points are indicated with white arrowheads.

**Additional file 2**: Vienna Moamin, folio 10v. The analyzed points are indicated with white arrowheads.

**Additional file 3**: Vienna Moamin, folio 19r. The analyzed points are indicated with white arrowheads.

**Additional file 4**: Vienna Moamin, folio 37r. The analyzed points are indicated with white arrowheads.

**Additional file 5** Vienna Moamin, folio 47v. The analyzed points are indicated with white arrowheads.

**Additional file 6** Self-built fiber optic probe: a) collimator 0.3 mm (latex), b) 0°/45°-component, c) xz-axial positioning mechanism, d) laser pointer for precise positioning, e) battery compartment, and f) switch for lasers on/off.

**Additional file 7** Comparison of the absorption index spectrum of a light blue colorant on folio 37r (red) with the IRUG reference spectra (obtained in transmission mode) azurite MP00001 (blue) and lead white MP00107 (yellow).

**Additional file 8** Left: Comparison of the Raman spectrum of a green colorant on folio 47v (red) with reference spectra of orpiment (yellow) and indigo (blue). Right: FORS spectrum from the same measurement point in comparison with an indigo reference.

**Additional file 9** Comparison of the Raman spectrum of a brown colorant on folio 1r (red) with an iron oxide brown reference (blue)

**Additional file 10** Comparison of the Raman spectrum of a black colorant on folio 10v (red) with a carbon black reference (blue).

**Additional file 11** Comparison of the absorption index spectrum of a blue colorant on folio 1r (red) with the IRUG reference spectrum (obtained in transmission mode) calcium stearate OF00108 (blue).

**Additional file 12**: The original size of the image stitched in Photoshop in Fig. 11 is 3355 x 3355 px.

## Data Availability

NA.

## Abbreviations

CIMA: Centre of Image and Material Analysis in Cultural Heritage
FORS: Fiber optic reflectance spectroscopy
HRSM: Hochschul-Raum Struktur-Mittel
IRUG: Infrared and Raman Users Group
mp: Measurement point
r: Recto
rFTIR: Fourier transform infrared spectroscopy in the reflection mode
v: Verso
XRF: X-ray fluorescence
