# Supplementary figures and images for: Materials and techniques used for the “Vienna Moamin”: multianalytical investigation of a book about hunting with falcons from the thirteenth century
Source: Herit Sci. 2021 Jul 23;9(1):87. doi: 10.1186/s40494-021-00553-w (PMC8550665; doi:10.1186/s40494-021-00553-w)

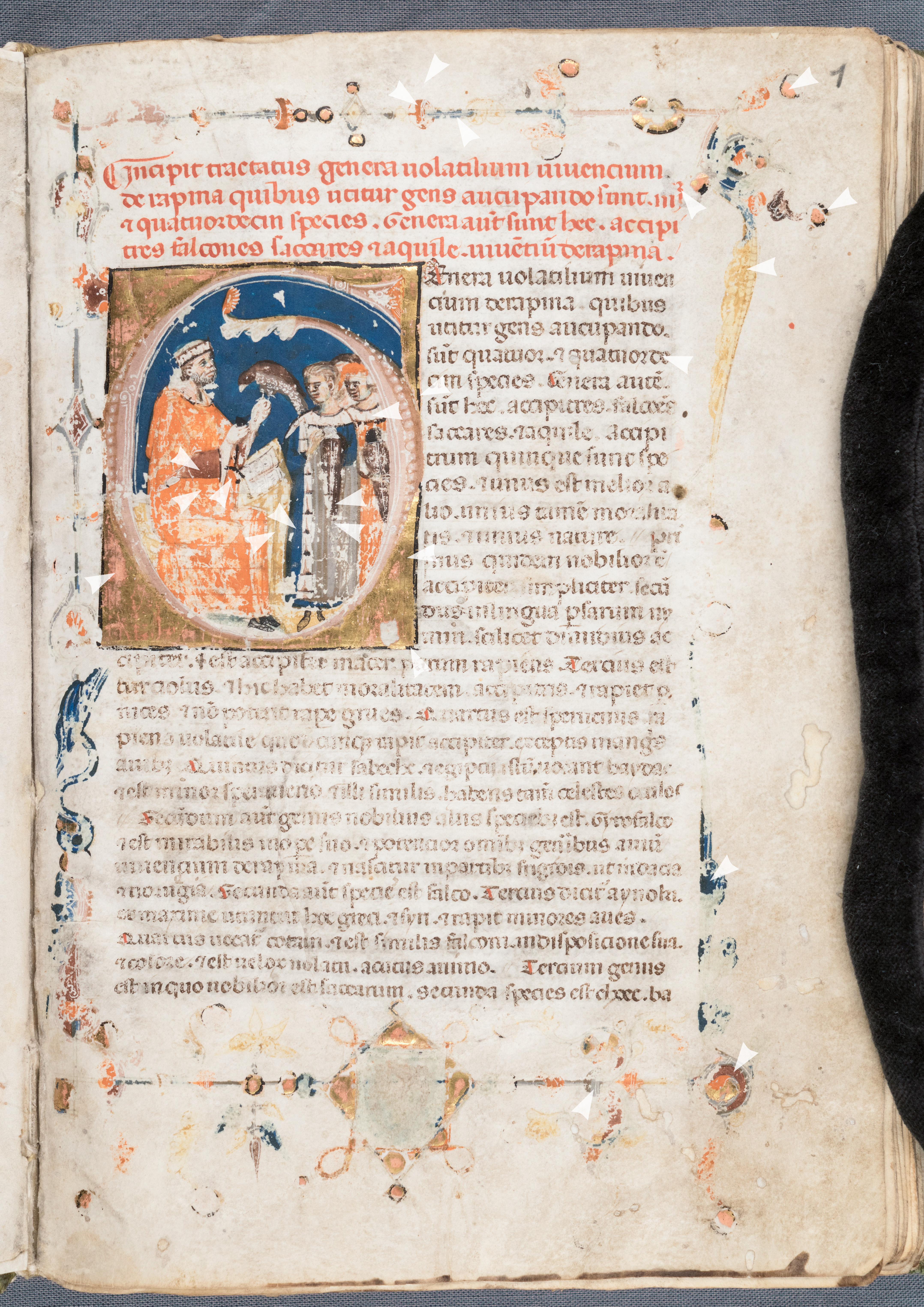

Supplement: Supplementary file 1 — Additional file 1 Vienna Moamin, folio 1r. The analyzed points are indicated with white arrowheads. [file 40494_2021_553_MOESM1_ESM.jpg]

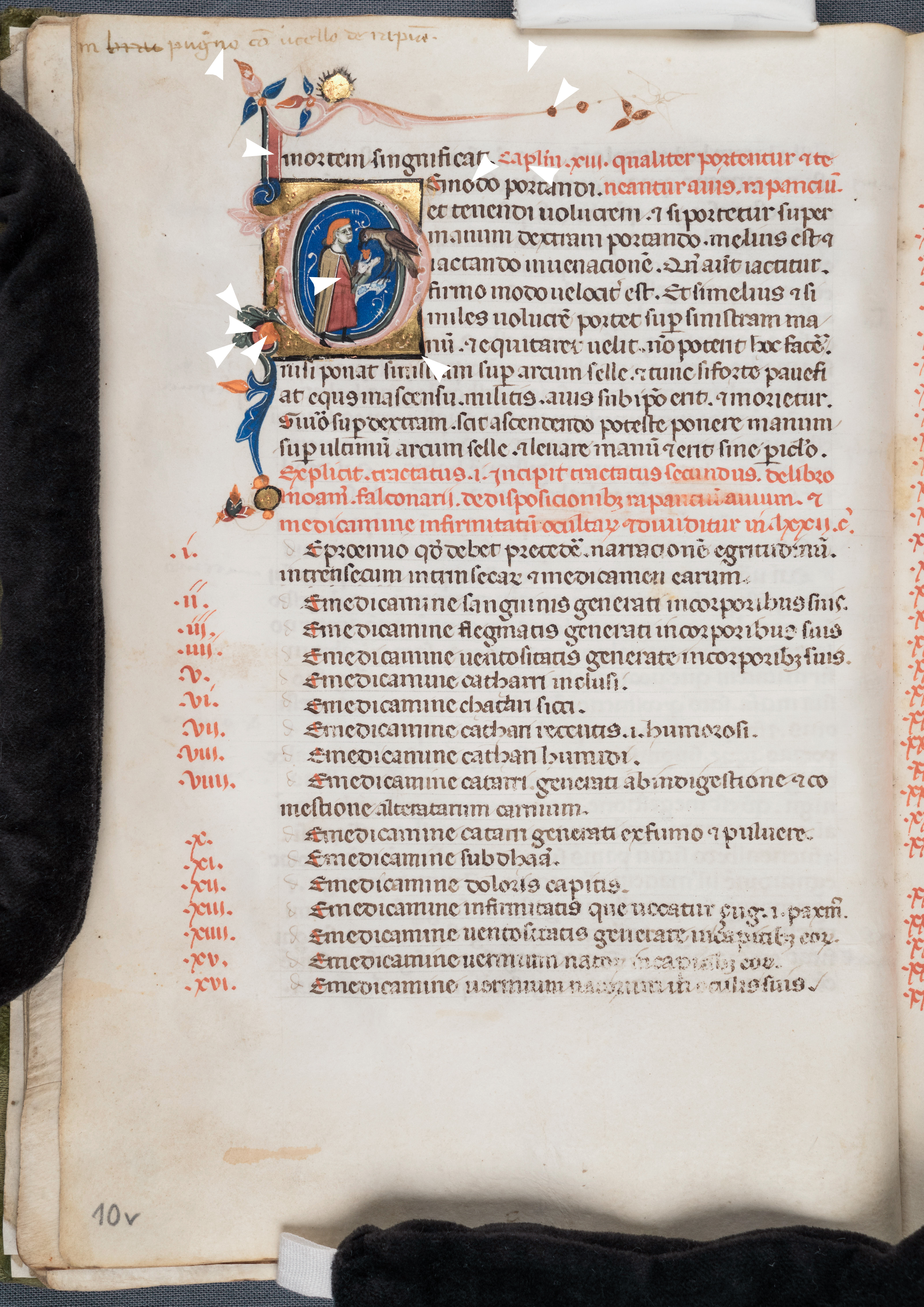

Supplement: Supplementary file 2 — Additional file 2: Vienna Moamin, folio 10v. The analyzed points are indicated with white arrowheads. [file 40494_2021_553_MOESM2_ESM.jpg]

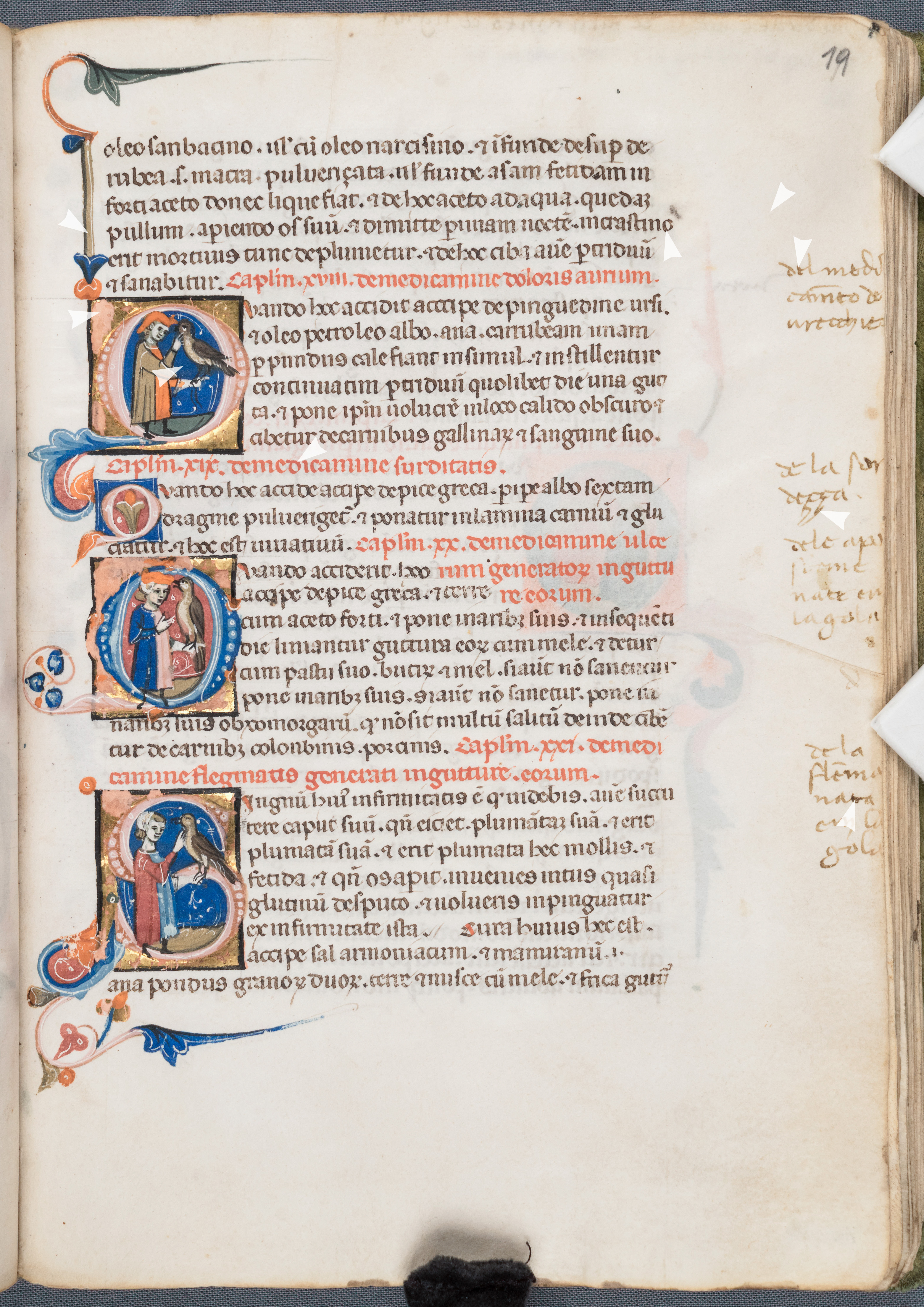

Supplement: Supplementary file 3 — Additional file 3: Vienna Moamin, folio 19r. The analyzed points are indicated with white arrowheads. [file 40494_2021_553_MOESM3_ESM.jpg]

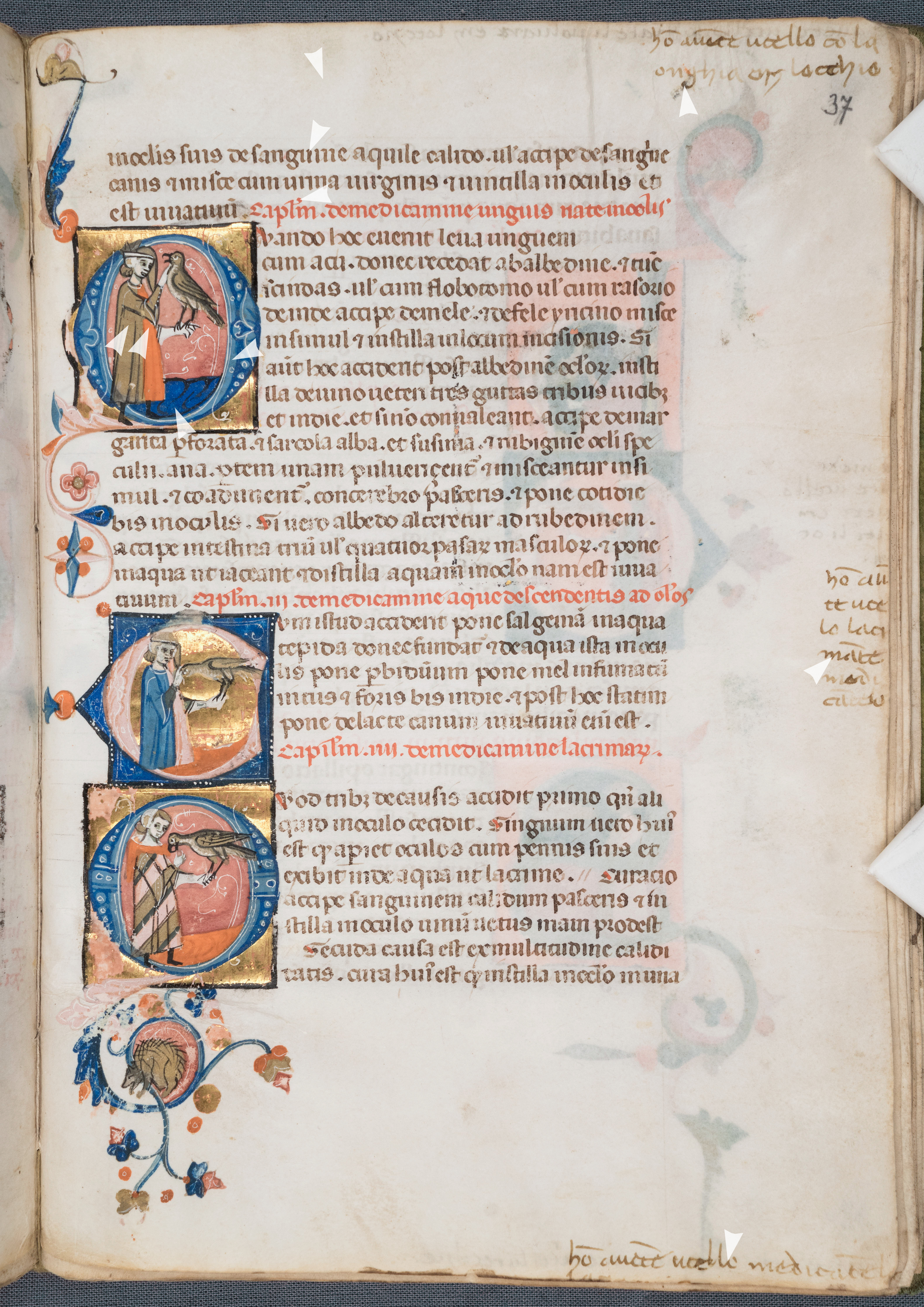

Supplement: Supplementary file 4 — Additional file 4: Vienna Moamin, folio 37r. The analyzed points are indicated with white arrowheads. [file 40494_2021_553_MOESM4_ESM.jpg]

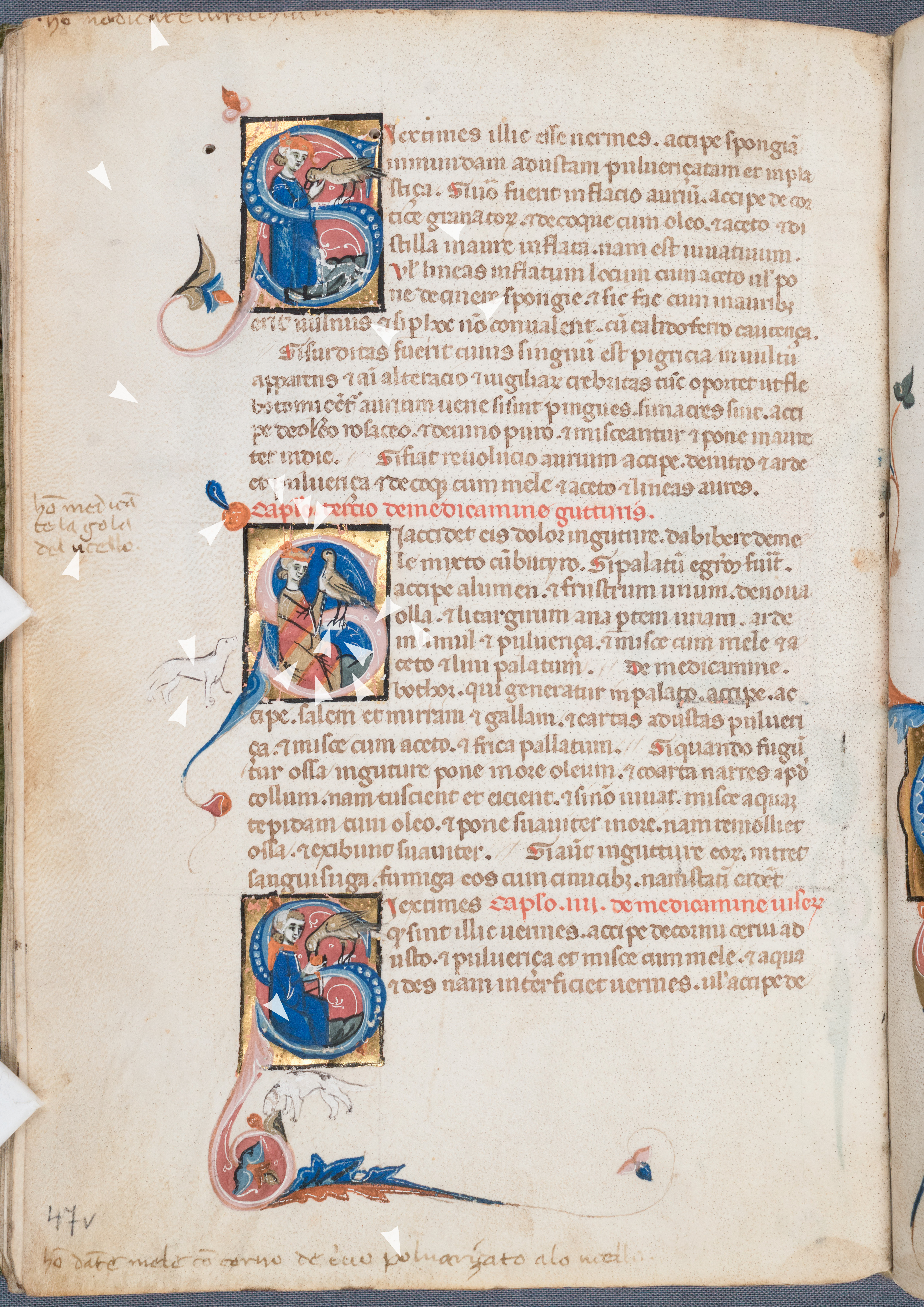

Supplement: Supplementary file 5 — Additional file 5 Vienna Moamin, folio 47v. The analyzed points are indicated with white arrowheads. [file 40494_2021_553_MOESM5_ESM.jpg]

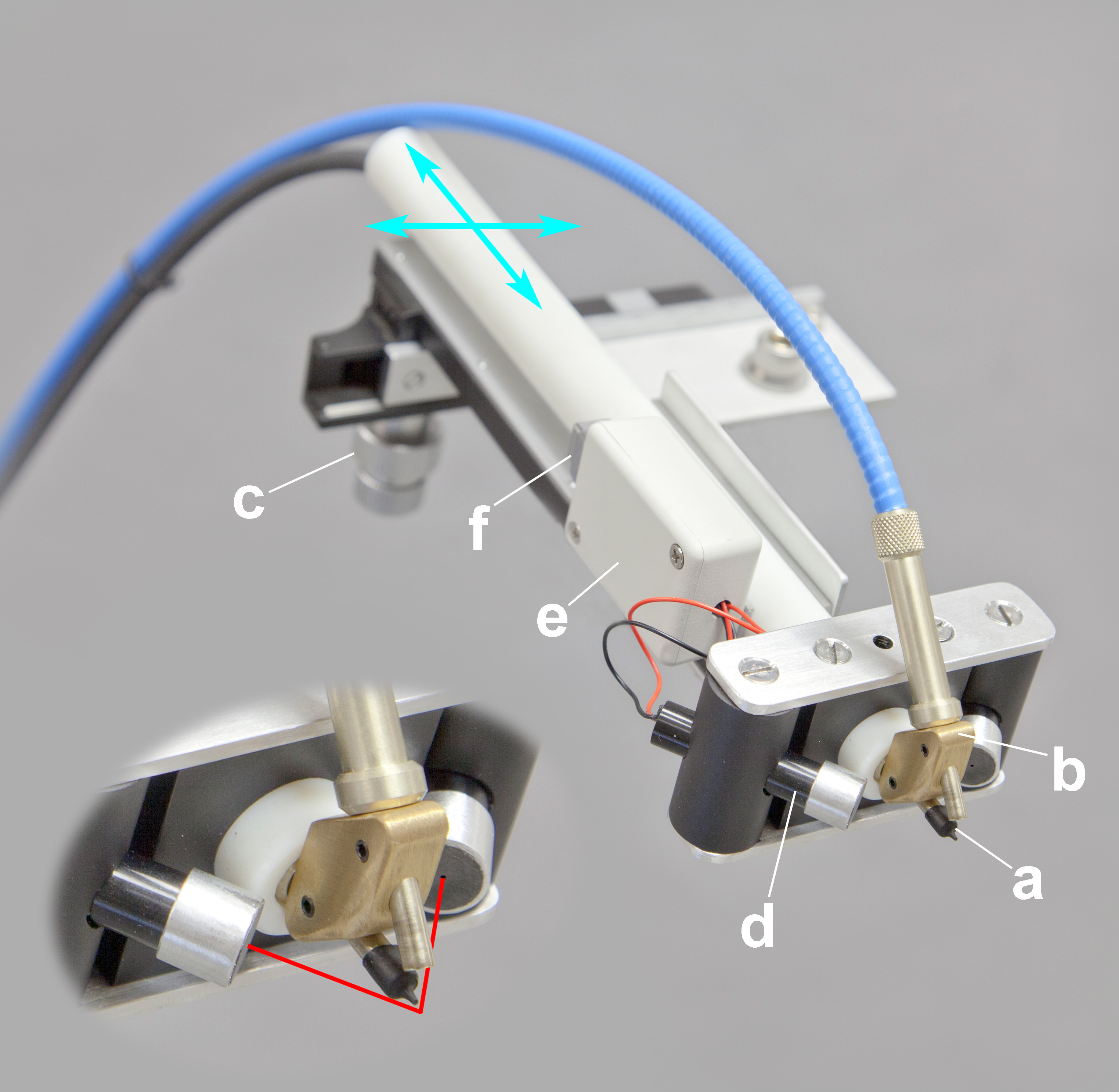

Supplement: Supplementary file 6 — Additional file 6 Self-built fiber optic probe: a) collimator 0.3 mm (latex), b) 0°/45°-component, c) xz-axial positioning mechanism, d) laser pointer for precise positioning, e) battery compartment, and f) switch for lasers on/off. [file 40494_2021_553_MOESM6_ESM.jpg]

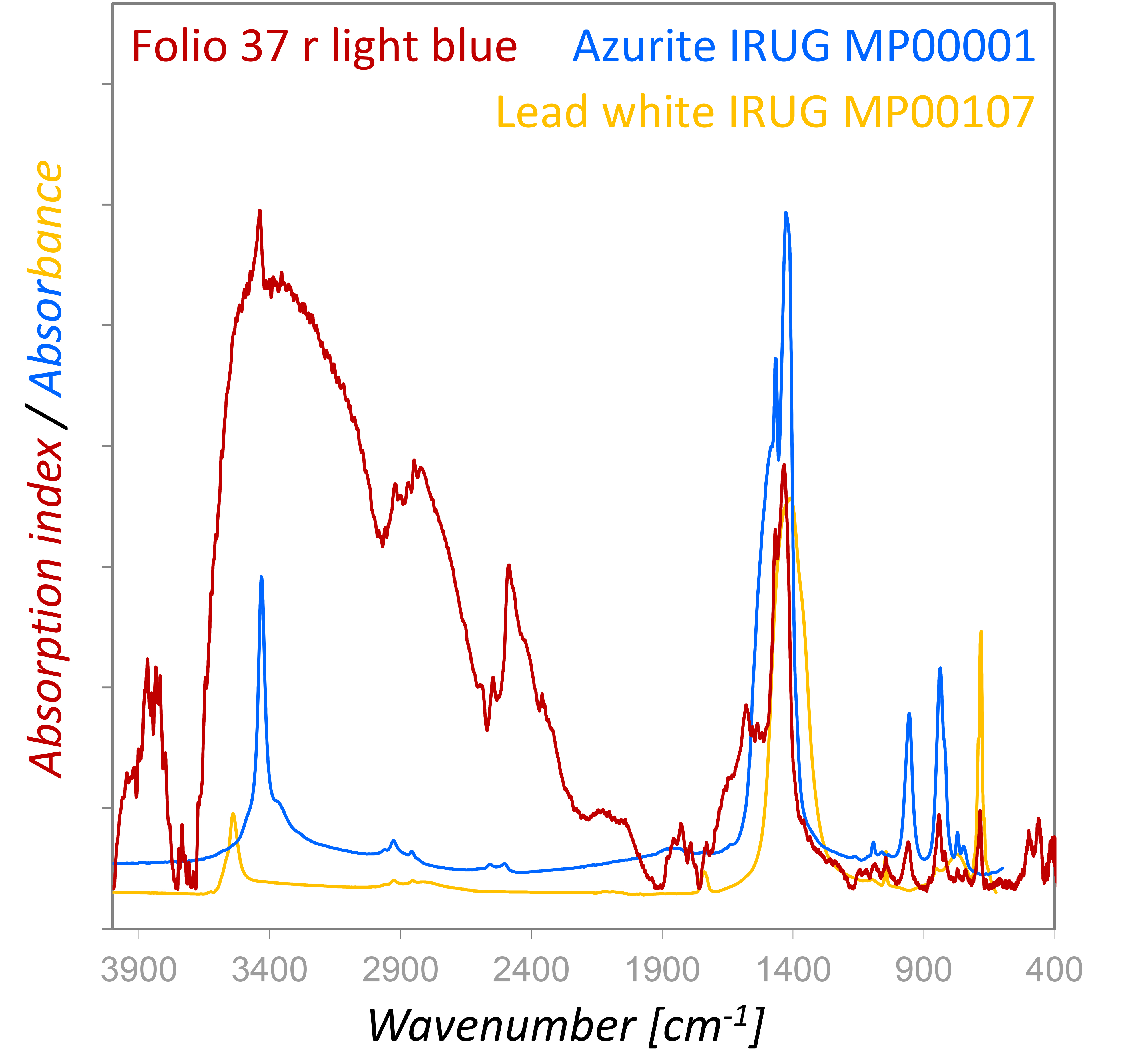

Supplement: Supplementary file 7 — Additional file 7 Comparison of the absorption index spectrum of a light blue colorant on folio 37r (red) with the IRUG reference spectra (obtained in transmission mode) azurite MP00001 (blue) and lead white MP00107 (yellow). [file 40494_2021_553_MOESM7_ESM.jpg]

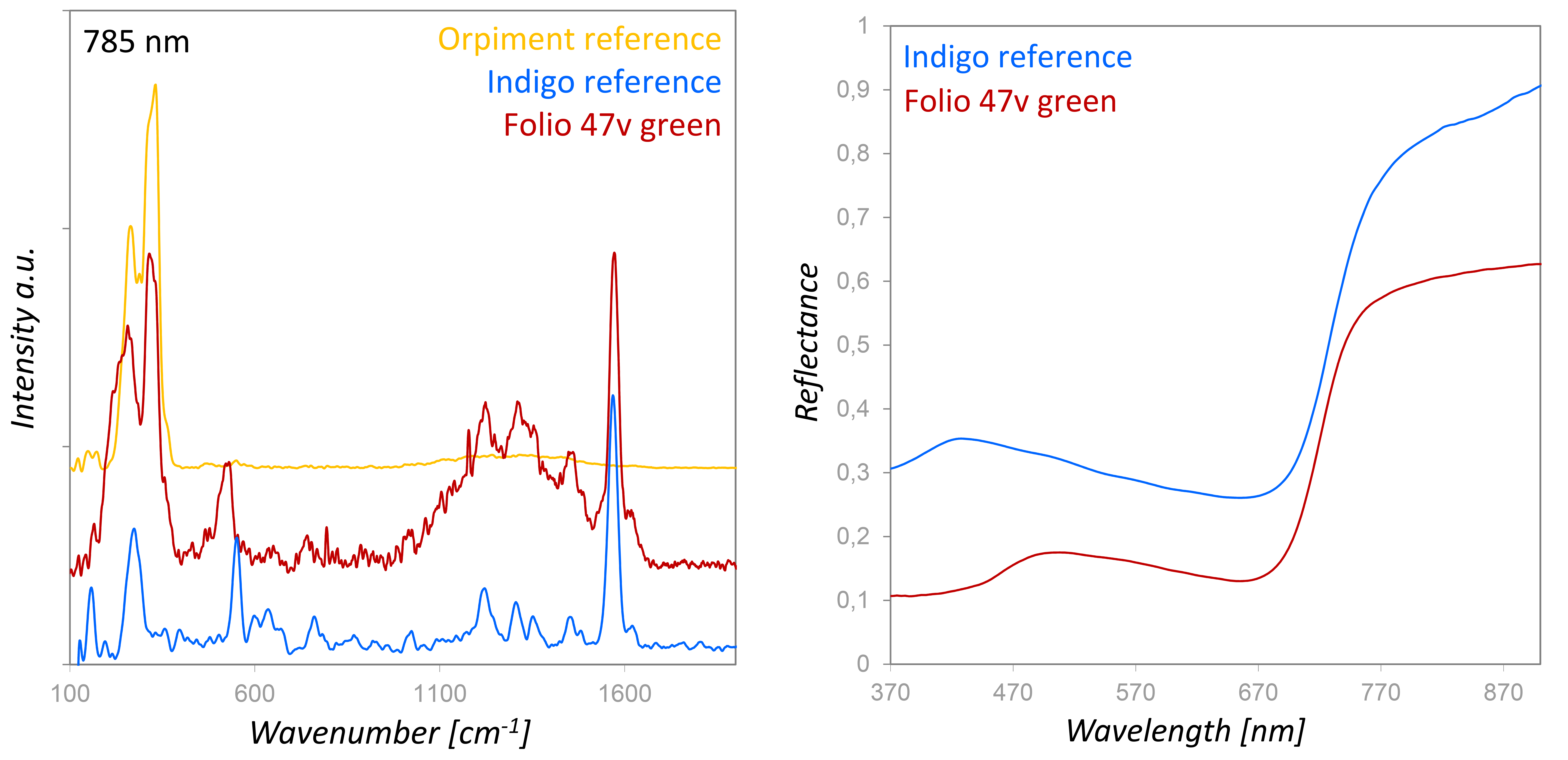

Supplement: Supplementary file 8 — Additional file 8 Left: Comparison of the Raman spectrum of a green colorant on folio 47v (red) with reference spectra of orpiment (yellow) and indigo (blue). Right: FORS spectrum from the same measurement point in comparison with an indigo reference. [file 40494_2021_553_MOESM8_ESM.jpg]

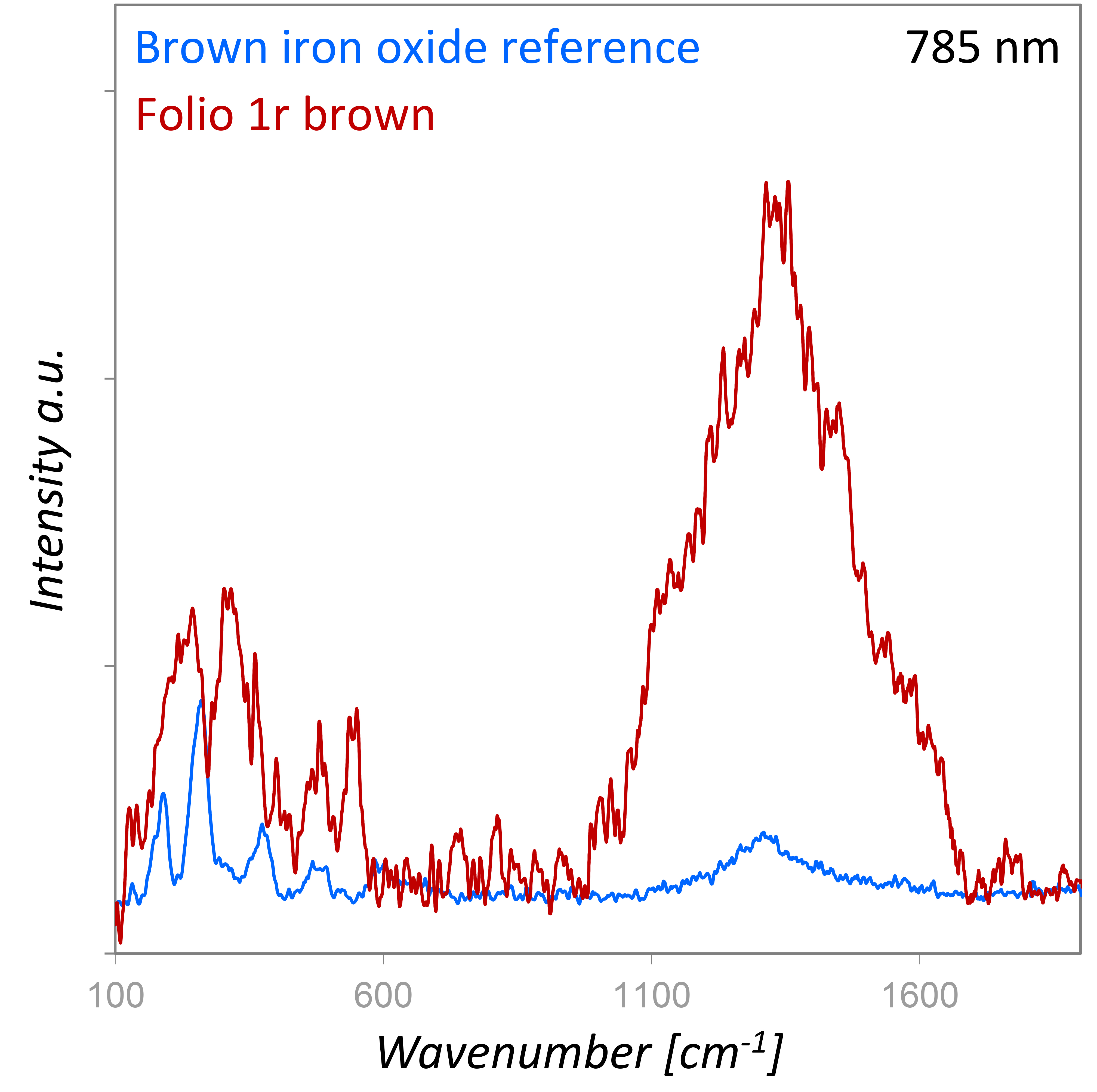

Supplement: Supplementary file 9 — Additional file 9 Comparison of the Raman spectrum of a brown colorant on folio 1r (red) with an iron oxide brown reference (blue) [file 40494_2021_553_MOESM9_ESM.jpg]

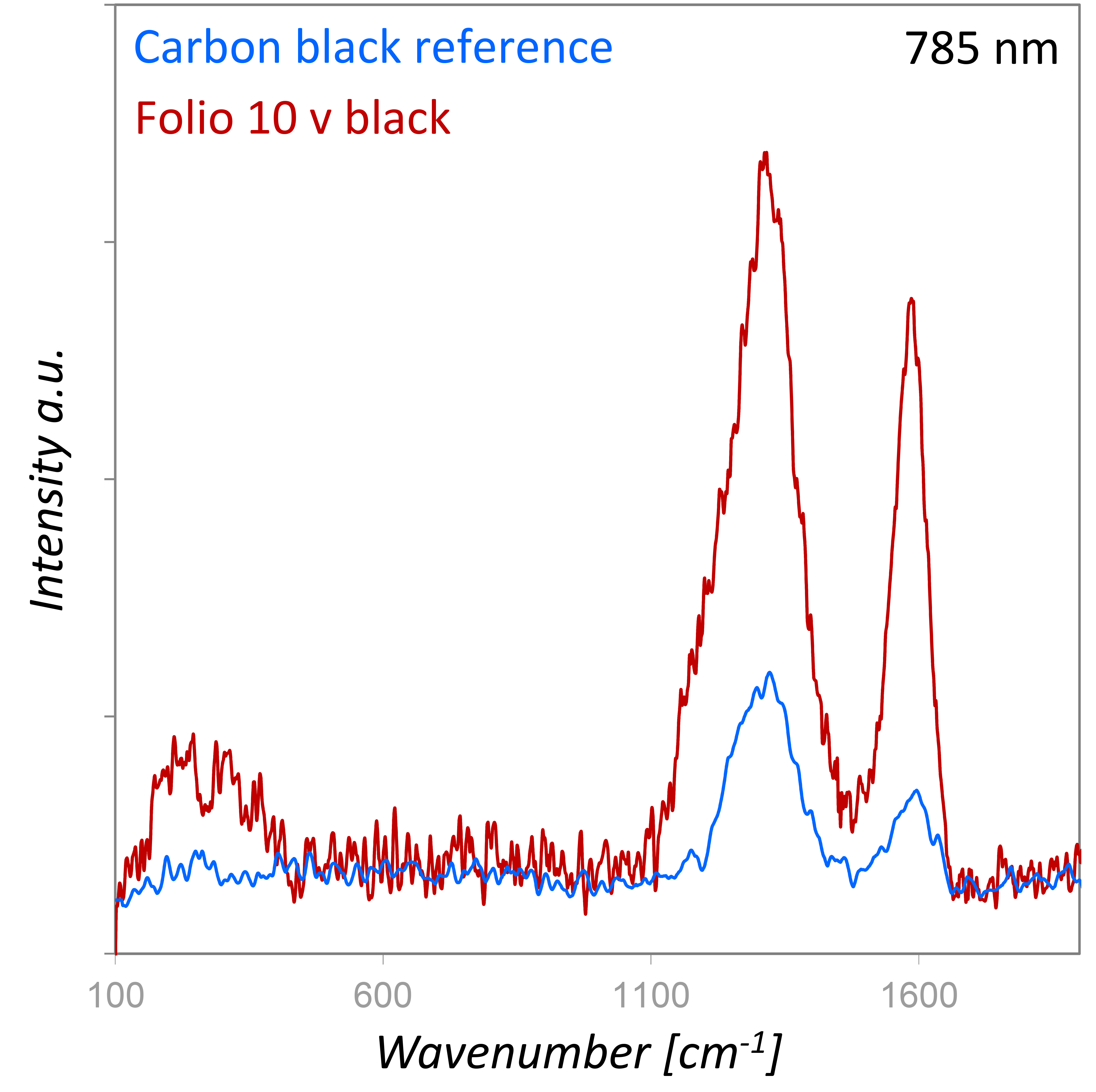

Supplement: Supplementary file 10 — Additional file 10 Comparison of the Raman spectrum of a black colorant on folio 10v (red) with a carbon black reference (blue). [file 40494_2021_553_MOESM10_ESM.jpg]

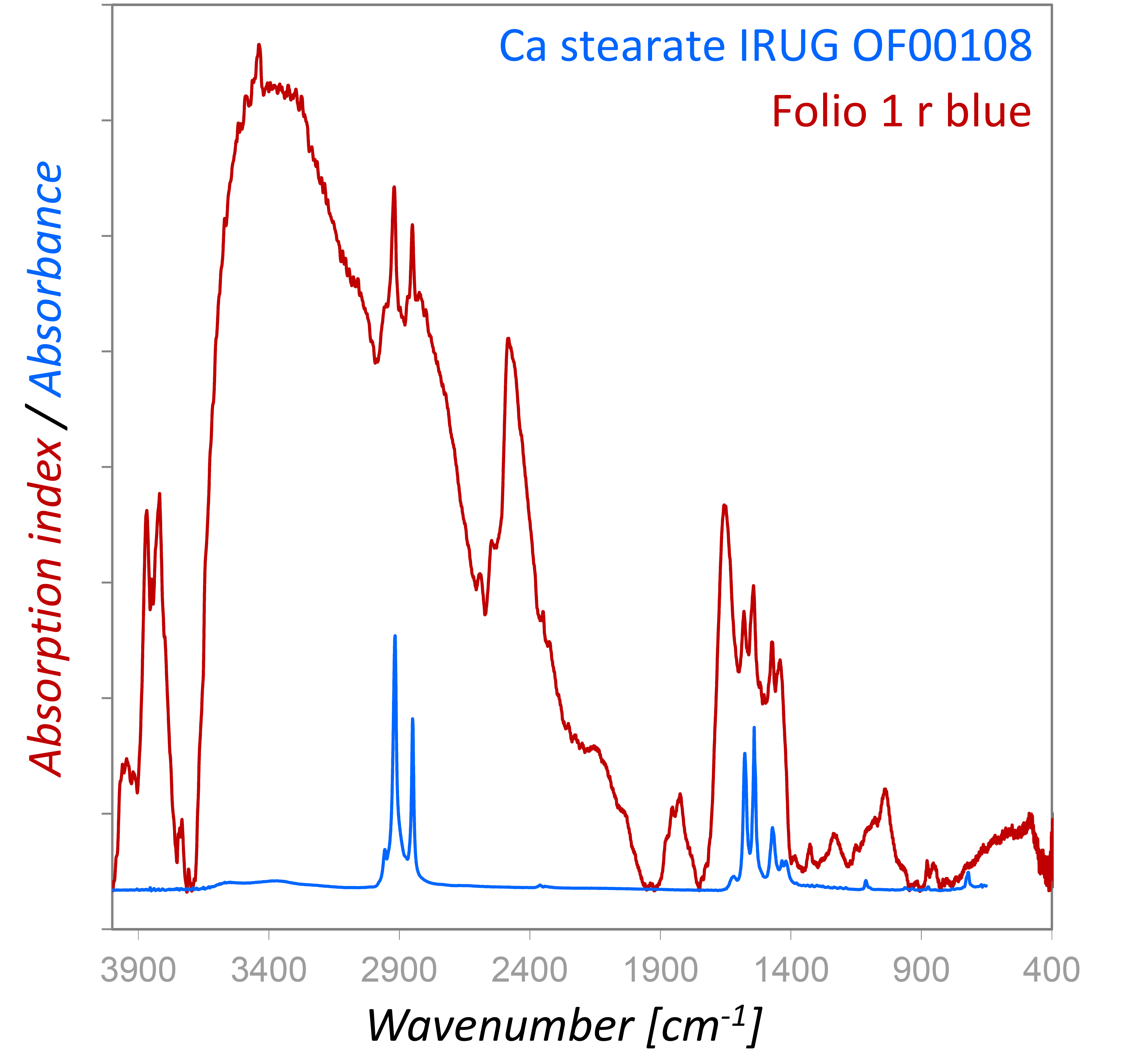

Supplement: Supplementary file 11 — Additional file 11 Comparison of the absorption index spectrum of a blue colorant on folio 1r (red) with the IRUG reference spectrum (obtained in transmission mode) calcium stearate OF00108 (blue). [file 40494_2021_553_MOESM11_ESM.jpg]

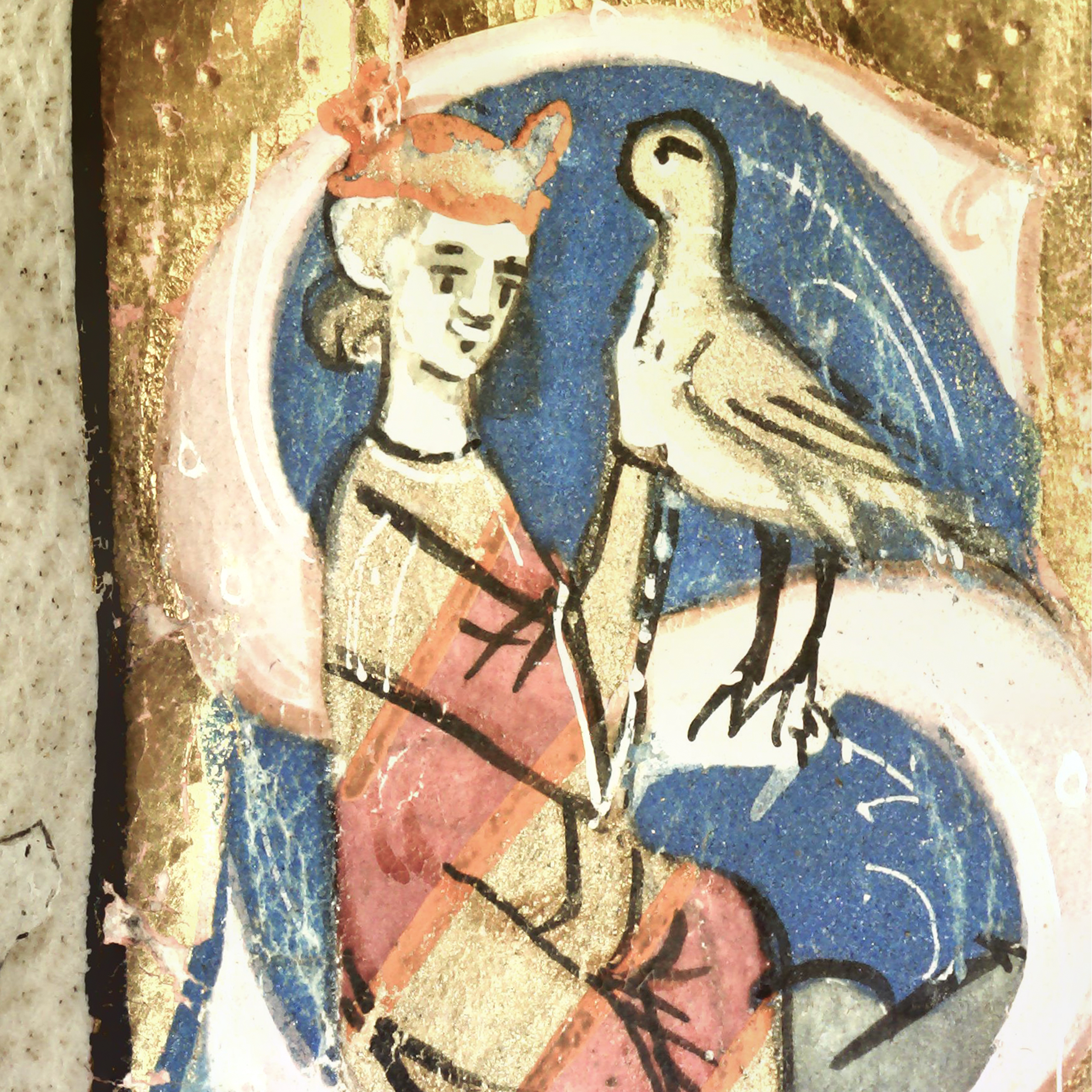

Supplement: Supplementary file 12 — Additional file 12: The original size of the image stitched in Photoshop in Fig. 11 is 3355 x 3355 px. [file 40494_2021_553_MOESM12_ESM.jpg]
